# The Transformation and Export of Organic Carbon Across an Arctic River‐Delta‐Ocean Continuum

**DOI:** 10.1029/2022JG007139

**Published:** 2022-12-12

**Authors:** J. Blake Clark, Antonio Mannino, Maria Tzortziou, Robert G. M. Spencer, Peter Hernes

**Affiliations:** ^1^ Ocean Ecology Laboratory Code 616.1 NASA Goddard Space Flight Center Greenbelt MD USA; ^2^ Goddard Earth Sciences Technology and Research II University of Maryland, Baltimore County Baltimore MD USA; ^3^ Department of Earth and Atmospheric Sciences The City College of New York The City University of New York New York NY USA; ^4^ Department of Earth, Ocean and Atmospheric Science Florida State University Tallahassee FL USA; ^5^ Department of Land, Air and Water Resources University of California, Davis Davis CA USA

**Keywords:** land‐ocean continuum, carbon cycle, arctic, biogeochemical modeling, climate change

## Abstract

The Arctic Ocean is surrounded by land that feeds highly seasonal rivers with water enriched in high concentrations of dissolved and particulate organic carbon (DOC and POC). Explicit estimates of the flux of organic carbon across the land‐ocean interface are difficult to quantify and many interdependent processes makes source attribution difficult. A high‐resolution 3‐D biogeochemical model was built for the lower Yukon River and coastal ocean to estimate biogeochemical cycling across the land‐ocean continuum. The model solves for complex reactions related to organic carbon transformation, including mechanistic photodegradation and multi‐reactivity microbial processing, DOC–POC flocculation, and phytoplankton dynamics. The baseline DOC and POC flux out of the delta from April to September 2019, was 977 and 536 Gg C (∼80% of the annual total), but only 50% of the DOC and 25% of the POC exited the plume across the 10 m isobath. Microbial breakdown of DOC accounted for a net loss of 168 Gg C (17% of delta export) within the plume and photodegradation accounted for a net loss of 46.6 Gg C DOC (5% of delta export) in 2019. Flocculation decreased the total organic carbon flux by only 6.4 Gg C (∼1%), while POC sinking accounted for 63.3 Gg C (10%) settling in the plume. The loss of chromophoric dissolved organic matter due to photodegradation increased the light available for phytoplankton growth throughout the coastal ocean, demonstrating the secondary effects that organic carbon reactions can have on biological processes and the net coastal carbon flux.

## Key Points

- Total organic carbon export out of the delta to the ocean from April to September 2019 was 1.5 Tg C, 65% of which was dissolved organic carbon
- 50% and 25% of the total delta export of dissolved and particulate organic carbon crossed the 10 m isobath into the coastal ocean
- The breakdown of riverine organic matter increases light for phytoplankton growth in the surface ocean 100 s of kilometers into the ocean

## 1 Introduction

The Arctic Ocean carbon cycle is substantially influenced by large and highly seasonal riverine inputs due to the relatively small, enclosed ocean basin and large land mass (McClelland et al., 2012). Particulate and dissolved organic carbon (POC and DOC) exported by these rivers constitutes a mean annual export of 34–38 Tg C of DOC (Holmes et al., 2012; Manizza et al., 2009) and 5.8 Tg C POC (McClelland et al., 2016), which fuels photochemistry and microbial communities in estuarine and near coastal environments. Arctic rivers have a high yield (load normalized to watershed area) of DOC and POC rivaling even tropical rivers (Raymond & Spencer, 2015). The amount of water entering the Arctic from riverine systems has been increasing over the last 50 years in multiple systems with long‐term records (e.g., Feng et al., 2021; McClelland et al., 2006; Peterson et al., 2002). Therefore, if organic carbon concentration to river flow relationships remained relatively stable, organic carbon export from rivers to the coast has also increased. However, the total flux from the lower river reaches flowing through the deltaic systems and beyond the low salinity plume waters is much less certain. This is due to the difficulty in quantifying the river‐delta‐ocean water transport and organic carbon transformational processes, among other biogeochemical processes. In addition, Arctic deltas can act as a source or sink of organic carbon, changing the net flux and composition of the organic carbon that is exported to the ocean (Emmerton et al., 2008; Kipp et al., 2020). New modeling techniques leveraging valuable but sparse field data are needed to better characterize this transfer of reactive carbon to the coastal environment.

The organic carbon that is exported from rivers to the ocean is variable in composition and source and depends largely on the hydrology and geomorphology of the rivers (Lynch et al., 2019). Terrigenous DOC is generally enriched in chromophoric dissolved organic matter (CDOM), which is a reliable tracer of soil and vascular plant derived DOC such as lignin phenols (e.g., Johnston et al., 2021; Mann et al., 2016; Spencer et al., 2008). The POC pool varies substantially across river systems with estimated mean radiocarbon ages ranging from 2,000 to 5,500 years while DOC is generally young (Barnes et al., 2018; Campeau et al., 2020; Karlsson et al., 2016) although recent radiocarbon evidence shows permafrost DOC mobilization in the Mackenzie River (Schwab et al., 2020). The total POC export is roughly an order of magnitude smaller than DOC (Holmes et al., 2012; McClelland et al., 2016). While DOC, CDOM, and POC can have similar terrestrial sources such as vascular plant breakdown and soil leaching and mobilization, differing processes affect how each is transformed as it enters the river and is transported along the land‐delta‐ocean gradient. For instance, CDOM may undergo photodegradation which has been shown to not only decrease the total light absorption but generally increase the bioavailability of the DOC pool (e.g., Grunert et al., 2021; Moran et al., 2000; Ward et al., 2017) by release of small and more biodegradable molecules including ammonium, monosaccharides, and free amino acids (e.g., Bushaw et al., 1996; Helms et al., 2008; Tarr et al., 2001). POC sinks to and is resuspended from the bottom depending on particle size, water velocity, and concentration, and is also produced *in‐water* by phytoplankton growth and death. The *quantity* and *quality* of organic carbon exported to the coast is affected by the formation of POC by DOC through flocculation (Asmala et al., 2014; Sholkovitz, 1976), while further modifications are possible through simultaneous adsorption‐desorption of DOC onto abundant inorganic particles (e.g., He et al., 2016; Kipp et al., 2020). Further complicating this is the production of POC and DOC by phytoplankton growth and subsequent death, which is first‐order controlled by temperature, light, and nutrient availability, and the reworking of DOC by heterotrophic microbes (Grunert et al., 2021). All of these processes can drastically change along the land‐ocean continuum as CDOM is photodegraded and POC settles out of the water column.

There is also an interplay between CDOM light absorption and DOC bioavailability to microbial degradation that occurs during transit from the river deltas into coastal waters. CDOM absorption and photochemical efficiency increases exponentially with decreasing wavelength, but light propagation through the water column does not occur uniformly across the UV‐Visible portion of the spectrum. While POC and DOC generally have exponential absorption spectra, the other inherent optical properties (IOPs) of particulate backscattering and phytoplankton absorption have complex spectral shapes. As absorbing and scattering material is degraded, altered, or produced, the downward propagating light quantity and spectral distribution can change drastically. Thus, the light available to transform DOC into more bioavailable compounds as well as for phytoplankton primary production—two dominant in‐water processes in coastal carbon cycles—is subject to secondary feedbacks that are difficult to quantify with field and laboratory observations alone.

Clearly, the coastal Arctic organic carbon cycle is very complicated but as measurements and experiments in northern high‐latitude systems accumulate, the development and implementation of a coastal organic carbon cycle model has become viable. While previous modeling work has primarily focused on the coastal and open‐ocean phytoplankton and nutrient cycles (e.g., Babin et al., 2015), recently the importance and challenges with realistically estimating coastal carbon cycling in these systems has been further described (Le Fouest et al., 2018). The large delta systems and river plumes can also act as reactors and transformers of riverine organic carbon before it enters the ocean. These connected systems are dynamic and therefore need to be characterized from a physical and biogeochemical perspective as important regions of organic carbon transformation. The intensifying impacts of climate change on the Arctic makes the quantification of coastal carbon budgets and estimating the first‐order controls on the export of organic carbon within coastal systems of high scientific and socioeconomic value. This importance extends both regionally and globally, looking towards a substantially different Arctic ecosystem in the future.

To better estimate coastal organic carbon fluxes and cycling, a high‐resolution coupled coastal carbon cycle model was developed and implemented for the lower Yukon River, delta, and coastal ocean. The model represents the dominant organic carbon transformation processes described above and was implemented for the ice‐free months of 2019 when a field campaign with detailed organic carbon measurements allowed for model calibration and validation. The total organic carbon export, partitioned among distinct classes of DOC and POC, was estimated across the river delta mouths and out of the plume into coastal waters. In addition, the river delta water flow and flux distribution were estimated, and the model‐data comparison allowed for a first order assessment of potential deltaic inputs to the system. Carbon transformational processes such as photodegradation, microbial breakdown, and phytoplankton production were explicitly included in the model to estimate how organic carbon composition changes from the river, through the delta, and into the ocean. Model scenarios allowed for quantification of the relative impact of photodegradation, flocculation, and sinking/resuspension on the organic carbon flux and distribution across the delta‐plume‐ocean gradient.

## 2 Study Location and Methods

### 2.1 The Yukon River and Northern Bering Sea

**Figure 1:**
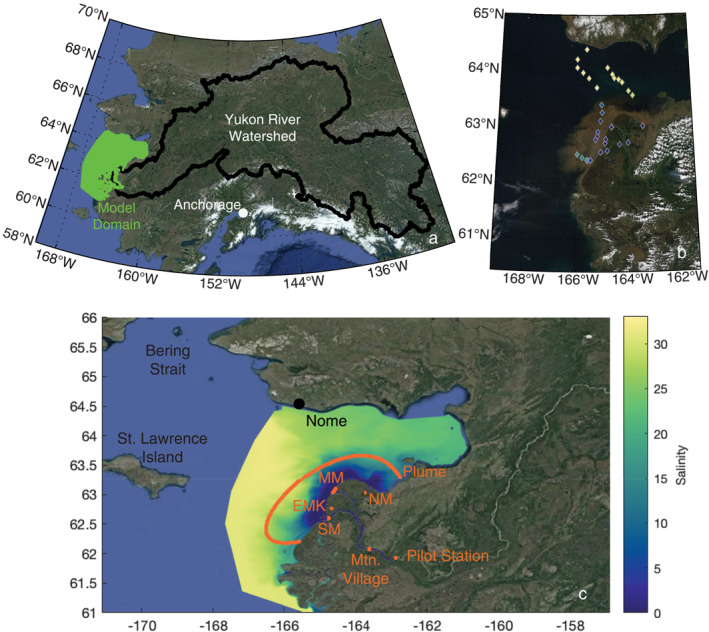
(a) The Yukon River watershed with the YukonFVCOM‐ICM model domain, which begins at Pilot Station and extends into Norton Sound toward the Bering Strait, (b) a true color satellite image acquired via NASA Worldview from 13 June 2019 depicting the sampling stations from the Yukon RSWQ2019 cruise that are colored by the measured surface salinity, and (c) the YukonFVCOM‐ICM model predicted salinity on 13 June 2019 overlayed with the transects used to calculate material flux from the biogeochemical model. Transect labels are Pilot Station, AK, Mountain Village, AK, the South Mouth (SM), the Emmonak Mouth (EMK), the Middle Mouth (MM), the North Mouth (NM) and the Plume, which is roughly defined by the 10 m isobath.

The Yukon River is the 5th largest river in North America (by volume) and the 5th largest river that contributes freshwater to the Arctic Ocean (Figure 1). Over 126,000 people live in the Yukon watershed, and it is the longest free‐flowing river in the world (Yukon River Inter‐tribal Watershed Council, 2021). The coastal currents where the Yukon flows into the ocean at the southwest edge of Norton Sound drive water northward through the Bering Strait where transport and freshwater content has increased over the last two decades (Woodgate, 2018). There is a substantial anti‐cyclonic eddy that increases residence time in the plume and moves water into meandering currents in Norton Sound (Clark & Mannino, 2022). The watershed is 830,000 km^2^ (Figure 1a) and contains 24% forest, 19% grassland and 60% discontinuous + continuous permafrost (HydroATLAS; Lehner et al., 2022; Linke et al., 2019). The coastal bathymetry is relatively shallow in the nearshore area with the model extending to ∼40 m depth toward the Bering Strait (Figure S1 in Supporting Information S1). Though the river doesn't flow directly into the Arctic, the entire watershed resides above 58.8°N latitude and is part of the Arctic Great Rivers Observatory (ArcticGRO; arcticgreatrivers.org). Like other northern high latitude rivers, there is growing evidence that total annual discharge has increased over the measurement record (Clark & Mannino, 2021; Feng et al., 2021), and the peak freshet is occurring earlier in the year (Novak et al., 2022). Long term records of discharge collected by the United States Geological Survey (USGS) at Pilot Station, AK, USA (∼200 km upstream from the coast), and measurements of many chemical parameters including total dissolved nitrogen (TDN), DOC, CDOM absorption, POC concentration, and suspended particulate matter (SPM) have been collected in all seasons beginning in 2003 under the Pan‐Arctic River Transport of Nutrients, Organic Matter, and Suspended Sediments (PARTNERS) project and from 2009 to present under ArcticGRO (Holmes et al., 2021). The long‐term records of chemical and discharge measurements at Pilot Station make the lower Yukon an ideal location for development and application of a river‐delta‐ocean biogeochemical modeling system for the Arctic.

### 2.2 Yukon River Hydrodynamic Model

The hydrodynamics of the lower Yukon River and coastal ocean are simulated using a high‐resolution regional implementation of the Finite Volume Community Ocean Model (FVCOM version 4.3) (Chen et al., 2003; Clark & Mannino, 2022). FVCOM is a three‐dimensional ocean circulation model developed to predict many physical properties of ocean systems on an unstructured triangular grid (Chen et al., 2003). It has been applied on multiple time and space scales to simulate Arctic Ocean physical properties including tidal transport in areas with complex coastlines and bathymetry (Chen et al., 2009, 2016). The Yukon River FVCOM (YukonFVCOM) was developed to estimate the transport and physical properties of water in the Yukon below Pilot Station, AK through the deltaic network and into the northern Bering Sea and Norton Sound (Figure 1) (Clark & Mannino, 2022). The model has demonstrated good ability at recreating temperature and salinity fields, validated with in situ profile data from 2017 to 2019 and satellite observations of sea surface temperature (SST) in 2004–2005 and 2015–2019. T, S, and sea surface height (SSH) are calculated at the nodes that connect each triangular element, and velocity vector components are calculated at element centers. The transport of tracers (T, S, and all simulated compounds in the biogeochemical portion of the model described in the next section) occur through tracer control elements (TCEs) between adjacent model triangular elements (Chen et al., 2003; Khangaonkar et al., 2017). TCEs are geometric areas between triangular elements that integrate the horizontal flow calculated at element centers and the tracer concentration at nodes between elements to calculate the mass flux among the triangular elements and nodes. Khangaonkar et al. (2017) developed methodology to accurately calculate the integrated water and salt flux across an arbitrarily defined transect within the model domain that was expanded to include all tracers within the biogeochemical model (Clark et al., 2020).

The model domain was developed using USGS HUC‐8 watershed mapping and contains 756,241 triangular elements connected at 435,240 nodes with 10 vertical sigma layers that make up a fixed proportion of the water column allowing the layer thickness to vary as the bathymetry changes. The model surface is forced by daily averaged North American Regional Reanalysis (NARR) (Mesinger et al., 2006) gridded weather data to calculate the air‐sea heat flux, the wind stress on the sea surface, and the downwelling irradiance. USGS daily average discharge and temperature is applied evenly over nine nodes at the upper boundary at Pilot Station. World Ocean Atlas 2018 (WOA18) monthly temperature (Locarnini et al., 2018) and salinity (Zweng et al., 2018) climatologies are used at the 389 open boundary nodes with TPXO SSH predictions (Egbert & Erofeeva, 2002) used to estimate the hourly variation in SSH at the boundary. The model domain included areas that were defined to be river channels or open ocean therefore the wetting and drying treatment available in FVCOM did not account for overland flow due to flooding and runoff. The physical model was run for three consecutive iterations in 2004 to generate the initial condition for April 1st, which was subsequently used for all years. The period of 1 April–15 May is considered spin up time because the river and ocean would typically be frozen during this period and the current model iteration doesn't dynamically represent sea ice. Our plans include year‐round simulations and dynamically predicted ice (and associated biogeochemical reactions) in the lower river and Norton Sound.

### 2.3 Dissolved and Particulate Organic Carbon and Inorganic Suspended Sediment

**Figure 2:**
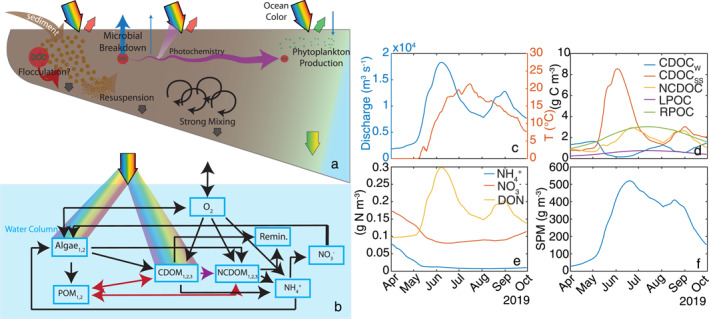
(a) A conceptual diagram showing the in‐water constituents and processes in the river plume with the colored arrows at the bottom representing the color of the in‐water light field and the waters entering and leaving the surface representing the downwelling and upwelling surface irradiance and radiance color, (b) a model flow diagram showing the model constituents and the pathways of mass transfer processes calculated in the model and the rainbow coloring indicating the constituents (algae and chromophoric dissolved organic matter; CDOM) that interact with the underwater light field and POM = particulate organic matter, NCDOM = non‐chromophoric dissolved organic matter followed by the time series of (c) river discharge and temperature, (d) organic carbon constituents including winter chromophoric dissolved organic carbon (CDOC_W_), spring/summer CDOC (CDOC_SS_), non‐chromophoric DOC (NCDOC), and labile and refractory particulate organic carbon (LPOC and RPOC), (e) dissolved nitrogen constituents, and (f) suspended particulate matter. Organic matter (DOM and POM) includes both carbon and nitrogen species. The river forcing time series were calculated using observations collected by the Arctic Great Rivers Observatory (Holmes et al., 2021), the USGS gauged river discharge, and the USGS LOADEST software (Runkel et al., 2004).

FVCOM has been coupled to multiple biogeochemical models that are used to calculate the transport and transformation of biochemical constituents in estuarine and coastal systems (Chen et al., 2013). A version of the carbon based 3‐D estuarine biogeochemical model, CE‐QUAL‐ICM, (Cerco & Cole, 1993) has been coupled with FVCOM in an offline manner to predict biogeochemical reactions that are important for coastal systems (Kim & Khangaonkar, 2012). Reaction formulations have been added to account for important chemical processes related to organic matter formation and transformation in estuarine systems in addition to simulating hyperspectral UV‐Visible light propagation and attenuation (Clark et al., 2020) making it suitable for use in an optically complex river outflow region. In this implementation, hereinafter referred to as YukonFVCOM‐ICM, NH_4_^+^, NO_3_^−^, six reactivity classes each of DOC and dissolved organic nitrogen (DON), inorganic suspended sediment (ISS), two phytoplankton classes, two reactivity classes each of POC and particulate organic nitrogen (PON), and dissolved oxygen are mechanistically represented (Figure 2a). In addition, the exchange of organic matter and nutrients across the sediment‐water interface and sediment diagenesis is dynamically calculated using the sediment flux model (SFM) (Brady et al., 2013; Di Toro, 2001; Testa et al., 2013) enhanced with dissolved organic matter formulations (Clark et al., 2017). This section will focus on the reaction formulations in the water column related to DOC, POC, SPM, and light attenuation but a full mathematical description of all biogeochemical components is provided in the appendices of Clark et al., 2020.

DOC has six reactivity classes, three chromophoric DOC (CDOC) and three non‐chromophoric DOC (NCDOC), where the CDOC undergoes photochemical reactions in addition to the breakdown by implicit heterotrophic microbes (Figures 2a and 2b) (Clark et al., 2019, 2020). CDOC is operationally defined as the mass of photoreactive DOC, rather than the typically measured CDOM which has units of light absorption. For this study, CDOC has been broken into three different classes based on source: marine CDOC (CDOC_M_), winter riverine CDOC (CDOC_W_), and spring/summer riverine CDOC (CDOC_SS_) and important parameters can be found in Table 1. Equation 1 shows the general DOC reaction formulation where the change in DOC mass over time at node *i* and layer *j*, dDOC d*t* ^−1^ (g C s^−1^), is equal to the physical transport among nodes and the biogeochemical source and sink processes. Physical transport (first term in Equation 1) is the summation of the product of the TCE edge (between surrounding nodes *m* to *n* and current node *i*), TCE edge normal velocity *U* (m s^−1^), the concentration of DOC ([DOC]; g C m^−3^) at surrounding nodes *m* to *n*, and the area (A; m^2^) of the face of the TCE between surrounding nodes *m* to *n* and current node *i*. Biogeochemical reaction terms follow where *κ*_b_ is the fraction of phytoplankton growth that is exuded as DOC, *μ* is the phytoplankton growth rate (s^−1^), which scales from a maximum value dependent on the relative light or inorganic nitrogen limitation (Clark et al., 2020), *B*_*i,j*_ is the concentration of phytoplankton (two types; g C m^−3^) at node *i* and layer *j*, *κ*_c_ is the heterotrophic microbial degradation rate (s^−1^) of the concentration of DOC ([DOC_*i,j*_]; g C m^−3^) which is modulated by the exponential temperature function *θ*_c_ (unitless), *κ*_p_ is the hydrolysis rate (s^−1^) of the concentration of POC ([POC_*i,j*_]; g C m^−3^) into DOC modulated by temperature function *θ*_p_ (unitless), *pd*DOC_*i,j*_ is the photochemical production or destruction of DOC (Clark et al., 2019) (g s^−1^), and *κ*_f_ is the flocculation rate (s^−1^) of riverine derived CDOC_W_ and CDOC_SS_ into refractory POC which is modulated by salinity, S (Equations 2a and 2b). All biogeochemical reaction terms are integrated by the volume of water surrounding at node *i* and layer *j*, *V*_*i,j*_, to yield the change in mass for each node and over time. A distinguishing feature of YukonFVCOM‐ICM is the ability to separate the optically active riverine CDOC components into different pools and reactivities based on measured optical properties and river seasonality. The derivation of the optical properties that determine the spectral and concentration dependent absorption of photons by CDOC are described further in the Text S1 in Supporting Information S1 and the inherent optical property (IOP) curves are displayed in Figure S2 in Supporting Information S1 and described briefly in Section 2.5 below.

**Table 1:** Model Parameters Related to the Dissolved Organic Carbon (DOC) and Particulate Organic Carbon (POC) Dynamics From the Equations in the Main Text

Symbol	Definition	Value	Units
*κ*_b_	DOC exudation fraction of phytoplankton production	0.2	ND
*κ*_c_	Microbial degradation rate of labile, semi‐labile, and refractory DOC	0.025, 0.01, 0.001	d^−1^
*κ*_p_	Hydrolysis rate of labile and refractory POC to DOC	0.03, 0.006	d^−1^
*κ*_fmax_	Maximum CDOC_W_ and CDOC_SS_ flocculation rate to POC, baseline and flocculation scenario	0.0, 0.1	d^−1^
*S*_max_	Salinity where maximum flocculation occurs	1.0	ND
*κ*_c1_	Shape parameter for sub‐optimal salinity for flocculation	5.0	ND
*κ*_c2_	Shape parameter for super‐optimal salinity for flocculation	0.25	ND
*ν*_B_	Implicit phytoplankton grazing rate for phytoplankton 1 and 2	1.5, 1.5	m^3^ g C^−1^
*f*_B_	Fraction of phytoplankton death that becomes labile and refractory POC	0.1, 0.5	ND
*w*_p_	Labile and refractory POC sinking velocity	1.0, 0.4	m d^−1^
*w*_max_	Maximum inorganic suspended sediment sinking velocity	2.0	m d^−1^
*κ*_ISS_	Inorganic sinking velocity half saturation concentration	51.0	g m^−3^
*τ*_crit_	Critical stress threshold for particle resuspension	0.005	Pa
*M*_*τ*_	Stress specific particle mass resuspension flux	1.0 × 10^−5^	g m^−2^ s^−1^ Pa^−1^

*Note.* All model parameters and equations can be found in Clark et al. (2020).

The paired flocculation formulations (Equations 2a and 2b) create a bell‐shaped curve where flocculation rapidly increases as salinity increases toward the salinity of maximum coagulation, *S*_max_, and declines once *S*_max_ is exceeded based on the curve‐shape parameters *κ*_c1_ and *κ*_c2_ and the flocculation rate (*κ*_f_) peaks to *κ*_fmax_ at *S*_max_. A figure of the curve implemented in the flocculation scenario can be found in Figure S3 in Supporting Information S1. The bottom layer of the model has an additional term that adds in the flux of DOC across the sediment‐water interface which is not shown in Equation 1 but is described in detail in Clark et al. (2017). In the baseline scenario, the flocculation rate is set to 0.0 as pending experimental results from the Yukon outflow will determine the net flocculation rate and dependence of flocculation on salinity and other physical factors. The flocculation model and parameterization lacks physical forcing such as turbulence due to the lack of experimental evidence from Arctic rivers. Future experiments of the dependence of flocculation on both ionic and turbulent processes will allow for a more complete mechanistic representation of DOC‐POC phase change interactions. Initial results presented here show a good overall model output‐data comparison for total DOC concentration across the plume without the inclusion of a flocculation term. However, a simple DOC‐POC flocculation scenario was tested to see how this likely reaction may impact the flux and composition of DOC and POC beyond the river delta into the coastal ocean.

(1) $$\frac{dDOC_{i,j}}{dt}=\sum_{m=1}^{n}U_{m}\left[DOC_{m}\right]A_{m,i}+\left(\kappa_{b}\mu B_{i,j}-\kappa_{c}\theta_{c}\left[DOC_{i,j}\right]+\kappa_{p}\theta_{p}\left[POC_{i,j}\right]+pdDOC_{i,j}-\kappa_{f}\left[DOC_{i,j}\right]\right)V_{i,j}$$

(2a) $$if\;S<S_{\max}\;then\;\kappa_{f}=\kappa_{fmax}e^{\left[-\kappa_{c1}{\left(S\;-\;S_{\max}\right)}^{2}\right]}$$

(2b) $$if\;S\geq S_{\max}\;then\;\kappa_{f}=\kappa_{fmax}e^{\left[-\kappa_{c2}{\left(S_{\max}\;-\;S\right)}^{2}\right]}$$

(3) $$\frac{dPOC_{i,j}}{dt}=\sum_{m=1}^{n}U_{m}\left[POC_{m}\right]A_{m,i}+\left(\nu_{B\;}f_{B}\theta_{B}B_{i,j}^{2}-\kappa_{p}\theta_{p}\left[POC_{i,j}\right]+\kappa_{f}\left[DOC_{i,j}\right]\right)V_{i,j}+\frac{w_{p}\left(\left[POC_{i,j-1}\right]-\left[POC_{i,j}\right]\right)}{Z_{j}}V_{i,j}+M_{R}$$

(4) $$If\;\tau_{i}>\tau_{crit}\;then\;M_{R}=\frac{M_{\tau }\left(\tau_{\iota }-\tau_{crit}\right)}{Z_{b}}V_{i,b}$$

POC is separated into biolabile and refractory (LPOC and RPOC) fractions that are classified by the rate of POC hydrolysis into DOC, *κ*_p_ (Table 1). Equation 3 represents the change in POC mass over time, dPOC d*t* ^−1^ (g C s^−1^) which is modeled similarly to DOC, but the biogeochemical processes contain two different terms in addition to the previously described hydrolysis of POC to DOC and flocculation of DOC to POC that have the opposite sign in the POC differential equation. The main autochthonous source of POC is from phytoplankton predation and death, represented by an implicit second order predation rate, *ν*_B_ (m^3^ g C^−1^) of the square of phytoplankton concentration B at node *i* and layer *j*, scaled by a temperature control function *θ*_B_ (unitless). The predation formulation prevents phytoplankton from growing too rapidly and the formulation becoming unstable and is parameterized according to the prey clearance rate of zooplankton in an estuarine environment (Clark et al., 2020; Kimmel et al., 2006). The sinking of POC via a constant settling velocity *w*_p_ (m s^−1^) between the POC in the layer above, [POC_*i,j−*1_] (g C m^−3^) and the POC in the current layer, [POC_*i,j*_], is integrated by the thickness of the current layer, Z_*j*_ (m). The final term in Equation 3 is the resuspended mass of POC, *M*_R_ (g s^−1^) that occurs when the bottom shear stress at node *i, τ*_*ι*_ (Pa), exceeds the critical shear stress for resuspension, *τ*_crit_ (Pa) (Equation 4). POC and ISS are resuspended at the same mass rate and shear stress (Table 1).

(5) $$\frac{dISS_{i,j}}{dt}=\sum_{m=1}^{n}U_{m}\left[ISS_{m}\right]A_{m,i}+\frac{w_{ISS}\left(ISS_{i,j-1}-ISS_{i,j}\right)}{Z_{j}}V_{i,j}+M_{R}$$

(6) $$W_{ISS}=W_{\max}\frac{\left[ISS\right]}{\left[ISS\right]+k_{ISS}}$$

The change in mass of ISS over time, *d*ISS *d*t^−1^ (g s^−1^) (Equation 5), has the same advection, sinking, and resuspension formulations as POC and there are no internal (not boundary forcing) chemical or biological sources of ISS to the water column. However, ISS has a concentration dependent settling velocity that is a Michaelis‐Menten like saturating function (Equation 6). This sets the maximum settling velocity, *W*_max_ (m s^−1^) to be approached as concentrations exceed the half saturation concentration, *k*_ISS_ (g m^−3^) and the effective settling velocity decreases as concentration declines toward 0 (Equation 6). This is a simple formulation to address the particle composition and concentration dependent settling velocity that likely occurs in a high‐sediment river environment (e.g., Nowacki et al., 2012), and it allowed for a much closer match of the SPM (ISS + POC) concentration to the data without introducing many sediment classes. The effective settling velocity of total SPM decreases as the organic content of particles increases from the delta into the ocean because the mean density of particles further away from the coast decreases.

### 2.4 River Forcing From Pilot Station

Measurements of POC, DOC, ISS (difference of SPM and POC), NO_3_^−^, NH_4_^+^, and DON (difference of TDN and NO_3_^−^ and NH_4_^+^) were collected at Pilot Station, AK beginning in 2003 and expanding to all seasons in 2009 (Holmes et al., 2021). After 2009, CDOM absorption spectra were measured which allowed for the estimation of CDOC concentration from the measurement data. All data are publicly available and updated as measurements are processed and can be found along with metadata on the ArcticGRO website (arcticgreatrivers.org). To estimate daily concentration of all measured and estimated biochemical constituents, the USGS Load Estimator (LOADEST) software program was used (Runkel et al., 2004). LOADEST builds a statistical model of measured concentration (calibration data) as a function of coincident river discharge (*Q*; m^3^ s^−1^) to predict mass flux (load) with observed *Q* (Figures 2c–2f). Daily POC, ISS, NO_3_^−^, NH_4_^+^, and DON concentration were estimated directly using all available observations and the observed daily mean discharge at Pilot Station. The daily mean concentration (g m^−3^) was estimated by dividing load (g d^−1^) by river *Q*.

Riverine DOC was fractionated into its chromophoric components based on the CDOM absorption spectral properties and the concept of having a winter “base flow” CDOC and spring/summer “high flow” CDOC. This was done by separating the spectra by month and grouping by season following Holmes et al. (2012) where winter is November–April, spring is May–June, and summer is July–October. Next, DOC was modeled as a linear function of CDOM absorption at 300 nm (*a*_300_) to find the initial estimate of the NCDOC as the intercept of the ordinary least square regression (Figure S4 in Supporting Information S1). Seasonal CDOC was then estimated as the difference between DOC and NCDOC while mass specific CDOC absorption spectra, a*_CDOC_(*λ*) (m^2^ g C^−1^), were calculated by dividing each individual CDOM absorption spectra *a*_CDOM_(*λ*) (*n* = 50) by the associated CDOC concentration and averaging for the winter (a*_CDOCw_(*λ*)) and spring/summer (a*_CDOCss_(*λ*)). Equation 7 was then used to estimate the concentration of CDOC_W_ and CDOC_SS_ as a function a*_CDOC_(*λ*). The MATLAB function *lsqnonneg* was used to estimate CDOC_w_ and CDOC_ss_ for each DOC and CDOM measurement by iteratively estimating the concentration and optimizing the concentration to the measured *a*_CDOM_(*λ*). This procedure is similar to that for estimating the concentration of CDOC in a photodegradation model (Clark et al., 2019). The individual estimates of CDOC_w_, CDOC_ss_, and total DOC were then used as the calibration data in LOADEST to predict daily concentration for 2019 (Figure 2d). Finally, NCDOC was estimated as the difference between total DOC and CDOC_w_ and CDOC_ss_ and fractionated into labile (NCDOC_1_), semi‐labile (NCDOC_2_), and refractory (NCDOC_3_) by 3%, 9.7% and 87.3% following microbial incubation results of Clark and Mannino (2021) and Wickland et al. (2012) that exhibited low overall biological availability of Yukon River DOC.

(7) $$a_{CDOM}\left(\lambda \right)=a_{CDOCw}^{*}\left(\lambda \right)\left[CDOC_{w}\right]+a_{CDOCss}^{*}\left(\lambda \right)\left[CDOC_{SS}\right]+a_{CDOCm}^{*}\left(\lambda \right)\left[CDOC_{M}\right]$$

The input of delta lakes was parameterized using the river‐connected lake volume information detailed by Piliouras and Rowland (2020). The total lake volume input was parameterized as a single point source above the delta near Mountain Village with an assumed flushing rate of the total lake volume (0.647 km^3^) per year. Parameterized lake concentration was assumed to be that of the input from Pilot Station on the same day, an assumption that likely underestimates the concentration of CDOM and DOC but overestimates the concentration of TDN (Novak et al., 2022). The total lake volume was fractionally distributed following the same discharge pattern of Pilot Station over the 183‐day model period. In total, the lake volume input of 0.647 km^3^ is only 0.43% of the 150.5 km^3^ from Pilot Station and therefore likely had minimal impact on the total concentration and flux of material within the river delta. However, this volume input is very conservative and doesn't account for other potential and likely substantial sources of DOC such as precipitation induced runoff and groundwater. These external delta inputs will be better characterized with future measurements in the delta.

### 2.5 Spectral Light Attenuation Model and Parameterization of Inherent Optical Properties

Hyperspectral light attenuation by optically active material and the water itself is predicted at 5 nm intervals for 285–700 nm at every node and vertical layer in the water column using an IOP based light attenuation model. This allows for a unique assessment of the impact of optically active material on the quantity and spectral quality of light that propagates through the water column and is thus available for phytoplankton growth and photodegradation of CDOM. This is done by calculating the total absorption, *a*_t_, as the summation of the absorption due to water, *a*_w_, *a*_CDOM_, the product of the mass specific chl *a* absorption, a* *φ* (m^2^ mg chl *a* ^−1^) and chl *a* concentration, and the product of the mass specific absorption of SPM, a*_SPM_, (m^2^ g^−1^) and SPM concentration (Equation 8). The backscattering in the water column due to the water and particles is then combined with *a*_t_ using the semi‐analytical algorithm of Lee et al. (2013) to predict the spectral diffuse attenuation coefficient *k*_d_(*λ*)*. k*_d_(*λ*) is then used in the standard exponential Beer's‐Lambert's light attenuation formulation to calculate the spectral downwelling irradiance at each layer in the model. The mass‐specific absorption spectra were numerically optimized to replicate measured water‐leaving radiance from the Yukon region, specific details of which along with each IOP spectrum can be found in the Supporting Information S1.

(8) $$a_{t}=a_{w}+a_{CDOM}+a_{\varphi }^{*}\left[chl\;a\right]+a_{SPM}^{*}\left[SPM\right]$$

### 2.6 Open Boundary Forcing From Cruise Data and WOA18 and Weather Forcing From NARR

To provide the external boundaries for the YukonFVCOM‐ICM modeling system, all of the constituent concentrations are specified along the 389 nodes along the western boundary in the northern Bering Sea and surface irradiance and wind velocity are specified at each node in the surface. WOA18 NO_3_^−^ (Garcia et al., 2018b) and dissolved oxygen (Garcia et al., 2018a) concentration were extracted from the global 1° database and the closest grid points to each boundary node were identified. The monthly climatological values were then interpolated using the MATLAB function *griddatan* to the model nodes horizontally and vertically to the model layers to achieve a complete 3‐d boundary of each variable for each month. This is the same procedure done for temperature and salinity for the hydrodynamic portion of the model. The monthly climatologies were then specified as the field for the first day of each month and the ICM model linearly interpolates between forcing points to the model time‐step. Daily mean surface irradiance and wind velocity from the NARR grid points that are over the model domain were extracted and specified uniformly over the nodes. Wind velocity is used to calculate air‐sea dissolved oxygen flux (Ho et al., 2006) and irradiance is used in many state variable reaction terms previously described. The NARR shortwave irradiance product was scaled by a factor of 0.43 to only use the proportion that is in the UV‐Vis range and then specified to each wavelength by the black‐body spectral distribution of light.

The open boundary DOC, phytoplankton carbon concentration, NH_4_^+^, POC and ISS were specified using cruise data from the NASA ICESCAPE 2010 and 2011 field campaigns (Arrigo et al., 2010) and the BEST‐BSIERP field campaign on the United States Coast Guard Healey 0803 (HLY0803) in the Bering Sea in July of 2008 (Goes, 2008). These cruises provided high quality archived data on the NASA SeaBASS data hub that were near the open boundary of the model domain and are publicly accessible and quality‐controlled (Werdell et al., 2003). DOC and *a*_CDOM_(*λ*) from the ICESCAPE cruise stations south of 66°N (4 stations, 21 samples) were extracted and DOC was linearly modeled as a function of *a*_300_ (*r* ^2^ = 0.9), and the total NCDOC concentration was estimated as the intercept OLS regression (0.48 ± 0.05 g C m^−3^) (Figure S4 in Supporting Information S1). Marine CDOC (CDOC_M_) was then estimated as the mean of the difference between total DOC and estimated NCDOC and the CDOC_M_ mass‐specific absorption spectra (a*CDOC_M_) was calculated by dividing each *a*_CDOM_(*λ*) by CDOC_M_, similar to the river CDOC estimates.

Chl *a* was extracted from the two ICESCAPE cruise data sets and classified as north (<66°N from ICESCAPE, 15 stations at 4 depths, 60 samples) and from HLY0803 as south (>60°N and >−168°W from HLY0803, 2 stations at 10 depths). chl *a* was interpolated to a uniformly spaced depth over 1–50 m at 5 m intervals and converted to phytoplankton C with an average C:chl *a* ratio from the Bering Sea shelf of 52.5 (g C g chl *a* ^−1^) (Lomas et al., 2012). POC was measured in the ICESCAPE cruises and was calculated for use in the model by subtracting the measured estimated phytoplankton biomass from the measured POC concentration and PON was calculated from POC by conversion using the Redfield ratio. All vertically interpolated stations were then averaged to generate the mean vertical profile for each constituent from the cruise data. The vertical profile was then linearly interpolated to the 10 model layers for each boundary node giving a vertically resolved concentration field across the boundary. Finally, the concentrations were estimated to be constant in time because of the limited measurements available. ISS was estimated at 1.77 g C m^−3^ from the average of the marine stations from NASA RSWQ2019 field campaign (Mannino & Novak, 2021). All files related to model forcing including plots of each variable on the boundary can be found at https://portal.nccs.nasa.gov/datashare/yukonriver/Carbon/inputs.

### 2.7 Model Validation Data, Statistical Analysis, and Scenario Set up

Observations collected on three research expeditions in May–June of 2019 were used to tune and validate the model predictions for salinity, chl *a*, DOC, CDOM absorption, SPM, and POC (Mannino & Novak, 2021). Salinity and satellite‐derived SST were further validated over 7 years throughout Norton Sound in a previous publication (Clark & Mannino, 2022). Statistical analyses used to validate the model against the observations includes the coefficient of covariance (*R* ^2^), Nash‐Sutcliffe model efficiency (MEF), and root mean square deviation (RMSD) (Stow et al., 2009). Previous results using target diagrams indicate that physical predictions of temperature and salinity show good agreement across years within the river plume and across Norton Sound (Clark & Mannino, 2022).

Five model scenarios were conducted to assess the effects of physico‐chemical processes related to the transport and transformation of organic matter from the river delta into the coastal ocean. First the baseline scenario (Base) utilized the hand‐tuned best model solution as a reference for calculating fluxes and estimating other biogeochemical processes. Next, we ran scenarios where the photochemical reactivity via the apparent quantum yield (change in carbon per photons absorbed) was set to zero (NoPD) and doubled (2xPD) to assess the impact of photodegradation on the total flux of DOC within the model domain and the distribution of DOC between reactivity pools. One scenario was tested involving the POC sinking velocity where the POC sinking velocity was set to 0.0 for both LPOC and RPOC from the baseline values of 1.0 and 0.4 m d^−1^, respectively. Finally, a scenario (Floc) was run with the initial implementation of the DOC‐POC flocculation formulation with the maximal salinity dependent flocculation (*κ*_fmax_) rate set to 0.1 d^−1^. Another scenario was also included where the microbial degradation of DOC was set to 0 to estimate the overall impact of microbial degradation on total DOC flux. Fluxes in various lower river and delta locations and across the river plume (Figure 1c) were compared, and the spatial variability of DOC, POC, and the underwater light field were assessed in the varying scenarios.

## 3 Results and Discussion

### 3.1 Model Output and Observation Evaluation and Comparison

**Figure 3:**
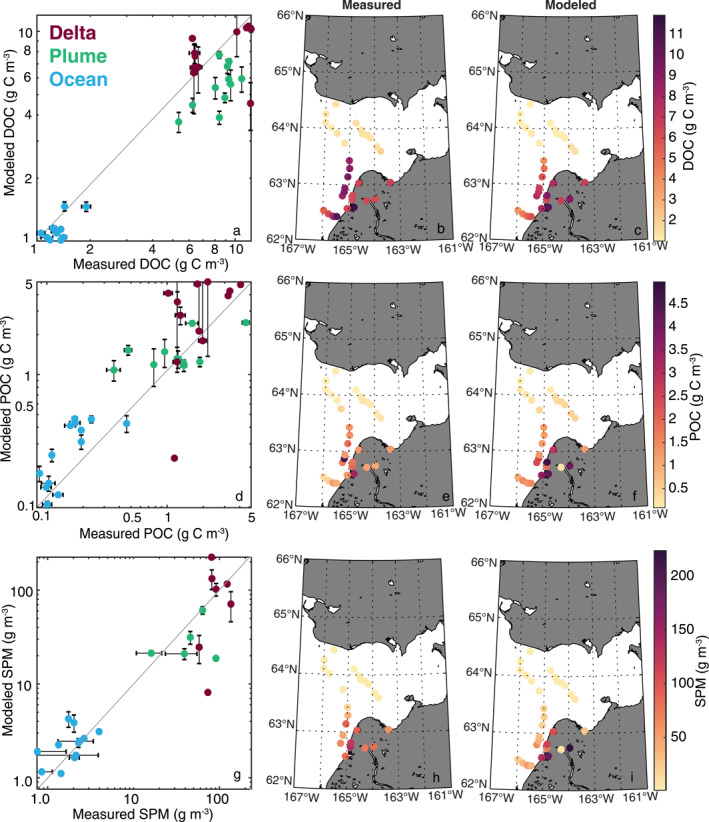
Dissolved organic carbon (DOC) concentration (upper row), particulate organic carbon (POC) concentration (middle row), suspended particulate matter (SPM) (lower row) for the closest match in time and space of the model predictions and the observations (Mannino & Novak, 2021). The vertical error bars in the first column represent ±1 standard deviation of the model output over a 36‐hr time period of the match and the horizontal error bars are ±1 standard deviation of replicate measurements, when available.

Observations of ocean physical and biogeochemical properties in 2019 in the Yukon delta and Norton Sound provided the baseline YukonFVCOM‐ICM model validation. In total, 36 observations of surface water properties spanning 30 May–29 June across the fresh to saltwater gradient allowed a relatively thorough comparison with the model predictions (Figure 3). DOC (*R* ^2^ = 0.70, MEF = 0.63) and POC (*R* ^2^ = 0.55, MEF = 0.03) were both well‐predicted across the gradient indicating that the first order inputs and processes were simulated reasonably **(**Figures 3a–3c and Figures 3d–3f) (Table 2). An MEF > 0 is indicative of a model with more predictive capacity relative to the mean, while an MEF = 0 is indicative of a model with the same predictive capacity as the mean of the observations. SPM (ISS + POC) was also captured within the model although fewer observations were available within the delta and on the southern transect due to a logistical issue in the RSWQ2019 field expedition (Figures 3g–3j). This indicates that the bulk processes of POC and ISS deposition and resuspension were well‐represented throughout the model domain and that the spatial distribution of SPM across the delta‐plume‐ocean was captured. Other important biogeochemical variables such as TDN and organic fraction of SPM were also well‐predicted, in addition to the physical variables of temperature and salinity (Table 2; Figures S5 and S6 in Supporting Information S1). Although accurately capturing processes in a model for this challenging environment was difficult, we feel confident that the results obtained here after over 80 model simulations are well‐suited for scientific analysis.

**Table 2:** Model‐Observation Comparison Statistics for Various State Variables, Definitions of Each Model Statistic Are Summarized in Stow et al. (2009)

	MEF^a^	R^2^ ^b^	RMSE^c^	Bias^d^	MPE (%)^e^	RI^f^
DOC	0.63	0.70	2.31 (g m^−3^)	1.03 (g m^−3^)	15.4	1.42
POC	0.03	0.55	1.11 (g m^−3^)	−0.46 (g m^−3^)	−68.0	1.99
SPM	0.14	0.34	32.6 (g m^−3^)	−5.58 (g m^−3^)	−16.1	2.03
TDN	0.38	0.78	0.07 (g m^−3^)	−0.06 (g m^−3^)	−47.1	1.57
chl *a*	−0.72	0.22	1.98 (mg m^−3^)	−0.17 (mg m^−3^)	−245.4	4.16
*a*_300_	0.54	0.78	12.7 (m^−1^)	8.8 (m^−1^)	43.2	2.20
POC:SPM	0.23	0.49	3.07 (%)	−1.54 (%)	−61.5	1.77
Salinity	0.96	0.96	2.42	−0.17	−89.5	10.1
Temperature	0.47	0.65	2.81 (°C)	1.58 (°C)	11.8	1.33

*Note.* All statistics were calculated in non‐log transformed space.

^a^ Nash‐Sutcliffe model efficiency (MEF) where values >0 indicate the model predictions are a more reliable predictor of a given observation relative to the mean of the observations.

^b^ Coefficient of variation (*R* ^2^) where 1 is a perfect match.

^c^ Root mean square error (RMSE).

^d^ Bias where a positive value indicates the model is less than the observations.

^e^ Mean percent error (MPE) where a positive value indicates the model is less than observations.

^f^ Reliability Index (RI) which is a measure of the relative factor of difference between model predictions and observations where a value of 1 is perfect.

Model‐predicted *a*_300_ showed strong covariance (*R* ^2^ = 0.78) with the observations, although the model tended to underpredict the magnitude of *a*_300_ (positive bias of 8.8 m^−1^) and had a larger mean percent error (MPE) than total DOC (43.2% vs. 15.4%). This indicates that the modeled total DOC pool was slightly depleted in CDOM relative to the observations. However, the forcing from Pilot Station of total DOC, CDOC_W_, and CDOC_SS_ was well‐matched with the observations with an estimated bias of −0.1%, 5.0%, and 19.3%. Indeed, the positive bias indicates that CDOC concentration from Pilot Station may even be slightly overestimated, on average. Therefore, a manual adjustment in the river forcing is not warranted to account for the difference between model predictions and observations from the delta and plume in 2019. This indicates that other sources of DOC that are highly enriched in CDOM exist within the delta, and particularly across the plume into the ocean, and therefore further observations and photodegradation experiments are required to characterize them throughout the ice‐free period. These additional and targeted observations and experiments within the delta and across seasons will lead to improvement in the simulation of CDOM in the delta and lower river and therefore a more accurate estimate of fluxes across the region in multiple years.

The resuspension and erosion of POC within the delta is a poorly characterized process but is likely important for the net POC flux from the delta to the ocean and can alter the overall composition of the total POC pool during delta transit. The model formulations related to POC settling and resuspension, in comparison to the real processes and other sophisticated suspended sediment models (e.g., Moriarty et al., 2017; Warner et al., 2008), are simplified. However, the model was successful at capturing the first order POC and SPM distribution, especially in the delta and river plume, indicating that complex processes (e.g., changing particle size and dynamic sinking velocities, changing source and composition of POC due to flocculation and erosion) can be captured in bulk with the simplified formulations. Moving the model from a static sinking velocity to a velocity that varied with concentration was a key formulation adjustment required to better capture both POC and SPM concentrations in the delta and ocean while keeping one ISS state variable. In reality, there are multiple orders of sizes of particles with varying compositions and densities that can substantially increase computational burden and parametrization. The new hyperbolic sinking velocity formula in YukonFVCOM‐ICM provided a good estimate while maintaining relative simplicity.

Model‐data chl *a* concentration covariance was not as strongly captured compared to the other biogeochemical variables, although there was little bias (−0.17 mg m^−3^) suggesting the overall average was captured. Elevated chl *a* in the river plume and low concentration in the ocean were particularly challenging to capture with the current model configuration, which was parameterized from phytoplankton physiological data collected in the Mackenzie River plume (Babin et al., 2015; Brugel, 2009). The phytoplankton community showed substantial diversity in pigment analyses collected from the RSWQ2019 cruise, and although light is extremely limited, measured chl *a* concentration within the plume and delta averaged 2.15 mg m^−3^. Photoadaptation of phytoplankton to low‐light conditions by increasing pigment concentration likely occurs. The inclusion of photoadaptation in the model requires a more sophisticated treatment of phytoplankton growth and variable pigment concentration. Instead we used a static C:Chl *a* ratio of 40.0 and 28.6, which is on the lower end of observations in Arctic systems (Babin et al., 2015). With extensive testing it was very difficult with the current formula and parameters to capture the spatial pattern in chl *a*. Targeted experiments and measurements of phytoplankton growth and physiology across the plume front would be invaluable to better understand how the model can be improved moving forward and characterize Arctic coastal ocean phytoplankton growth.

While we are unaware of measurements of net primary production in the coastal Yukon region, the model predictive capacity of water‐column chl *a* is similar to that of the other models in the Arctic Ocean (Lee et al., 2015). It is likely that attempting to represent such a large gradient across aquatic ecosystems with only two phytoplankton groups is insufficient as conditions shift from highly turbid, fresh, and nutrient rich to relatively clear, salty, and nutrient poor conditions in the span of tens of kilometers. However, the model's strong ability to simulate the variables related to light absorption and scattering (e.g., CDOM, POC, and SPM) suggests that with a more realistic construction and parameterization of phytoplankton within YukonFVCOM‐ICM, higher trophic levels and more complex metabolic processes can be included in the future.

### 3.2 Baseline Flux of Material Across the Delta and Into the Plume

**Figure 4:**
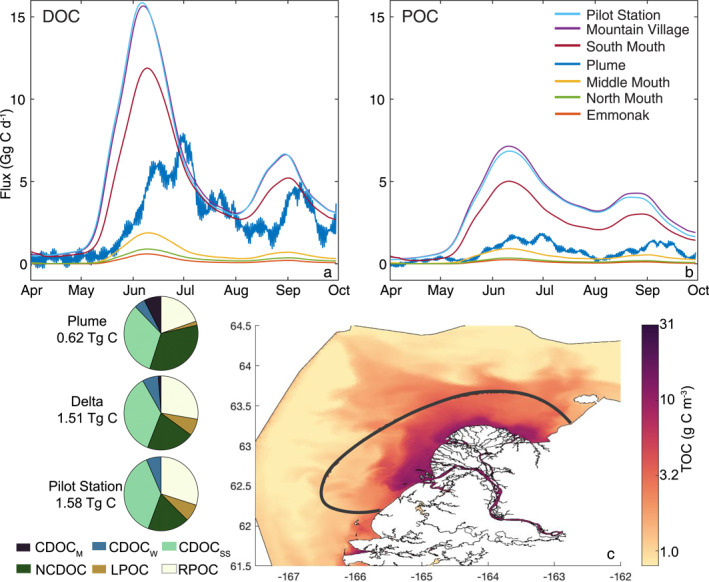
(a) The flux of dissolved organic carbon (DOC) and (b) particulate organic carbon (POC) across the seven locations identified in Figures 1c, and (c) a map of the surface total organic carbon (TOC) concentration on 30 June 2019 when the flux across the plume transect was greatest. The pie charts on the left show the relative distribution of the 6 classes of organic carbon defined by optical and biological lability, where CDOC_M_ is marine chromophoric (optically active) DOC, CDOC_W_ is riverine winter CDOC, CDOC_SS_ is riverine spring‐summer CDOC, NCDOC is non‐chromophoric (optically inert) DOC, LPOC is labile POC and RPOC is refractory POC. The total flux in Tg C is the time integrated flux at each location from 1 April to 30 September 2019.

The flux of DOC and POC within the river and delta followed the seasonal pattern of river flow, peaking with the primary freshet in June and the secondary pulse in late August and September (Figures 4a and 4b). In the river segment between the model input at Pilot Station and downstream at Mountain Village, before the delta begins, there was virtually no gain or loss of DOC (988 vs. 990 Gg C) (Figure 4) suggesting passive transport in this ∼55 km stretch of the lower river. The fraction of the DOC flux at Mountain Village that exited the delta at the delta mouths included as transects within the model was 99% which indicates the selected transects captured the vast majority of water flow and DOC passing from the river delta to the ocean‐ the net sources and sinks of total DOC within the delta are small even though composition may change. The south mouth (SM; Figure 1a) received most of the DOC (784 Gg C) and POC (425 Gg C) over the model period, equating to 79% and 68% of the total input from Pilot Station (Figure 4c). However, internal processes such as POC hydrolysis, sediment‐water column DOC fluxes, and phytoplankton exudation likely contributed to the total amount of DOC that left the delta, albeit a small amount due to the short (3–10 days) residence time. As discharge decreases into the summer, in‐water processes in the delta likely become more important on the net carbon pool (Novak et al., 2022), but a full carbon accounting budget with all model reaction terms integrated is needed to quantify this. The majority of the net flux of DOC from the river through the delta was captured, but the enrichment processes of the DOC pool with CDOM was not as well represented, as evidenced by the model‐data comparisons of *a*_300_ (Figures S6a–S6c in Supporting Information S1). Other processes such as soil leaching, bank erosion, groundwater flow, and flooding of the delta were not included in the model formulations but likely contribute to total delta inputs. A detailed analysis of the seasonal DOC concentration and CDOM absorption revealed that following a precipitation event there was an increase in DOC and CDOM in a delta tributary, indicating precipitation related inputs are a potentially important source that isn't captured (Novak et al., 2022). Comprehensive in situ characterization of these secondary sources is key to further advance source attribution of organic matter from the river delta into the coastal ocean.

The maximum daily flux of DOC (10 days moving average) of 15.9 Gg C d^−1^ at Pilot Station occurred at 0800 UCT on June 6, 15.7 Gg C d^−1^ 16 hr later at Mountain Village, and 11.9 Gg C d^−1^ at 0800 on June 9 at the South Mouth (Figure 4a). This indicates there was a ∼3‐day lag between the inputs at Pilot Station and the main outflow at the South Mouth during peak flow. The maximum DOC flux across the plume (Figure 1c) was 7.93 Gg C d^−1^ and occurred at 1600 on 30 June. The transport time of the main pulse from the river delta past the 10 m isobath to the coastal ocean and Norton Sound was ∼21 days. The 10‐day moving average smoothed out most of the tidal oscillations, but the semi‐diurnal and spring‐neap tidal variation is substantial (Figure S7 in Supporting Information S1). Physical oceanographic factors such as buoyancy‐driven circulation, spring‐neap tidal oscillations, and wind‐driven storms are overlayed in the oscillations of the plume flux time series (Clark & Mannino, 2022). The tidal stage must be a top‐level consideration during in situ sampling because there can be substantial variance in the concentration and flux across both semi‐diurnal and spring neap tidal periods in the coastal locations. Overall, 485 Gg C of DOC (49% of Pilot Station input) and 132 Gg C POC (22% of Pilot Station input) equating to 39% of the total organic carbon crossed the plume transect over the model period (Table 3).

**Table 3:** The Organic Carbon Flux Across the Plume and South Mouth Transects for Each Constituent That Makes Up the Total Dissolved Organic Carbon (DOC) and Total Particulate Organic Carbon (POC) Pool, in Gg C, From 1 April–30 September 2019

Scenario	CDOC_M_	CDOC_W_	CDOC_SS_	NCDOC	LPOC	RPOC	Total	% River input (total, DOC, POC)
Plume								
Base	46.1	29.2	202.9	206.8	10.9	121.2	617.1	39, 49, 22
NoPD	1.50	48.4	275.8	205.9	11.0	121.9	664.5	42, 54, 22
2xPD	75.5	18.6	157.1	207.8	10.8	120.2	590.0	37, 46, 22
NoSet	45.7	30.2	204.0	209.5	28.5	164.9	680.4	43, 49, 33
Floc	44.6	27.7	189.5	207.7	10.9	130.4	610.8	39, 47, 24
Delta								
Base	15.5	98.4	548.1	314.8	114.0	421.9	1,513	96, 99, 90
NoPD	10.8	101.8	556.2	314.7	114.0	421.9	1,519	96, 99, 90
2xPD	19.6	96.0	542.3	314.9	114.0	421.9	1,509	95, 98, 90
NoSet	15.5	98.5	548.1	314.9	114.5	420.9	1,512	96, 99, 90
Floc	15.5	98.4	547.5	314.8	114.0	422.0	1,512	96, 99, 90

There was a substantial shift in the overall composition of the DOC pool during transit from the delta to the plume, shown by the diagrams of the fraction of the total organic carbon for each location (Figure 4c). In the river and delta, the DOC pool was made up mostly of CDOC_W_ and CDOC_SS_ which was the prescribed input from Pilot Station; CDOC_M_ made up <1% and 1% (15.5 Gg C) of the total flux from Pilot Station and the delta. Across the plume transect, DOC made up a larger fraction of the total OC flux compared to the river transects (79% vs. 65%) due to the settling of riverine POC inshore of the 10 m isobath, and CDOC_M_ increased to 7% (46.1 Gg C) of the total flux. There is also a substantial increase in the relative proportion of NCDOC in the total flux from 21% in the delta to 34% across the plume while the proportion of CDOC_SS_, which is sourced from the river, decreased slightly in relative proportion (36%–33%) but substantially in the total mass flux (548.1–202.9 Gg C). The partitioning of organic carbon from the delta to the plume transect exhibited some emergent properties that are indicative of the underlying biogeochemical processes that affect each carbon pool: (a) The relative proportion of POC to DOC decreases substantially during transit into the ocean; (b) There was relatively less optically active CDOC due to photodegradation, although the DOC remaining was still enriched in river‐derived CDOC_SS_; (c) There was production of the more biologically labile CDOC_M_ by phytoplankton exudation and the photodegradation of riverine CDOC into CDOC_M_.

To assess the impact of microbial degradation on total DOC a simple model scenario was conducted without heterotrophic breakdown of DOC; all other model processes remained the same. This scenario showed that a loss of 168 Gg C can be attributed to the removal of DOC by microbial degradation within the delta and plume. This equates to 17% of the delta DOC flux and 35% of the DOC flux across the plume transect in the baseline scenario. It is notable that the scenario with no microbial degradation generated a plume DOC flux (653.3 Gg C) that was not closer to the delta DOC flux (990 Gg C). This is primarily due to the accumulation of DOC inshore of the 10 m isobath, but secondarily the potential loss of DOC from photochemical degradation and sediment‐water column exchange. To see the true impact of microbial degradation on the net plume flux, the model system necessarily needs to simulate multiple years to account for the loss of DOC during winter when river flow is near zero. Chemical evidence suggests that ∼50% of terrestrially‐derived DOC is remineralized within rivers and estuaries from the large Arctic Eurasian rivers (Kaiser et al., 2017), which strongly matches our estimate of 49.7% of DOC crossing the 10 m isobath into more open‐ocean waters. Chemical biomarkers and decay constants showed that biological degradation was the dominant removal process of terrestrial DOC in the dark‐incubated samples along the Eurasian shelf (Kaiser et al., 2017).

Recent experimental evidence shows the complex interplay between photochemical processes, CDOM absorption and degradation, and microbial transformation of DOC (Grunert et al., 2021). Photodegradation can lead to a substantial loss in CDOM absorption, but DOC transformation was quantitatively more important than photomineralization. After photodegradation, DOC was more bioavailable for microbial breakdown, but coupled photo‐ and microbial degradation showed complex transformations and humification processes, rather than a net enhancement of DOC breakdown via photodegradation (Grunert et al., 2021). The many other internal sources within the plume likely contribute to the total DOC pool that is transported from the mouths of rivers to the ocean. POC‐DOC phase change interactions (e.g., flocculation, Sholkovitz, 1976; Spencer et al., 2007) or adsorption‐desorption reactions (He et al., 2016; Hernes & Benner, 2003) during the strong increase of the ambient ionic strength may contribute to new source or sink processes of DOC. Indeed, we show below how a hypothetical flocculation term can shift DOC into the POC pool at the outflow of the river.

More uncertain is the contribution to the DOC pool from sediments (e.g., Burdige et al., 1992; Komada et al., 2013) and from phytoplankton growth and DOC exudation within the plume waters. Chl *a* concentrations were highest at the edge of the river plume (Figure S6 in Supporting Information S1), a phenomenon not well captured with the current modeling configuration. If these phytoplankton are actively growing, they would likely contribute to the total DOC pool, albeit DOC that is chemically distinct from terrestrial runoff which is typically enriched in plant‐derived compounds (Amon et al., 2012; Spencer et al., 2008). While the relative proportion of DOC in each class is indicative of some net production of marine DOC within plume waters, we can't further discern how much comes from photodegradation of riverine material or phytoplankton production from the baseline simulation alone. Having consistent measurements of terrestrial biomarkers across seasons, coupled with primary production measurements, would allow us to better discern the terminal source of DOC that is transported out of the plume.

### 3.3 Scenario Differences in Time and Space

#### 3.3.1 Photodegradation Scenarios

Photodegradation had virtually no effect on the net flux of DOC from Pilot Station through the delta with only *a* ±1% difference in the DOC flux from the baseline scenario for the NoPD and 2xPD scenarios across the delta (Table 3) which is unsurprising given the high turbidity and low light of the river. However, there was a change in the overall composition of the DOC pool between the different photo‐ and biological reactivity classes. The distribution of CDOC between the three classes changed due to photodegradation: there was an 81% greater flux (8.8 Gg C) of CDOC_M_ in the 2xPD versus NoPD scenario and 13.9 Gg C greater flux (2%) of CDOC_SS_ in the NoPD versus 2xPD scenario because CDOC_M_ is the main photodegradation product of riverine CDOC. Across the plume there was a 46.6 Gg C increase (9.6%) in the total DOC flux in the NoPD scenario, relative to the baseline, while the 2xPD scenario had a decrease in DOC flux of −26.0 Gg C (−5.4%) (Table 3). There was 46.6% (148.5 Gg C) more riverine CDOC that transported across the plume in the NoPD versus 2xPD scenario, with 74 Gg C more CDOC transported as CDOC_M_ in the 2xPD scenario (Table 3). There was a distinct shift in the distribution of the CDOC among the three pools with the NoPD scenario maintaining the bulk of the concentration in the riverine CDOC classes, while the 2xPD scenario shifted a greater proportion of CDOC toward the more biologically reactive CDOC_M_ class.

Photodegradation can substantially affect how terrestrial DOM is processed by the microbial community in the coastal Yukon (Grunert et al., 2021) and increases the overall bioavailability of DOM in Arctic rivers (Kellerman et al., 2019; Ward et al., 2017). Other environmental controls such as temperature, nitrogen availability (Clark & Mannino, 2021; Wickland et al., 2012), and microbial community composition also likely exert control on the degradation of the total CDOM and DOC pools. An increase in the overall microbial availability of the DOC pool due to photodegradation has been observed in other coastal systems with significant terrestrial and wetland input and is an inherent feature of the photodegradation module employed here (e.g., Logozzo et al., 2021; Miller et al., 2002; Moran et al., 2000). The shift toward more biological reactivity in the 2xPD scenario led to a greater microbial breakdown of DOC within the river plume, especially because direct photomineralization to CO_2_ (rather than changes in DOC composition and reactivity) is parameterized to be orders of magnitude lower than photobleaching between CDOC classes and into NCDOC (Clark et al., 2019).

**Figure 5:**
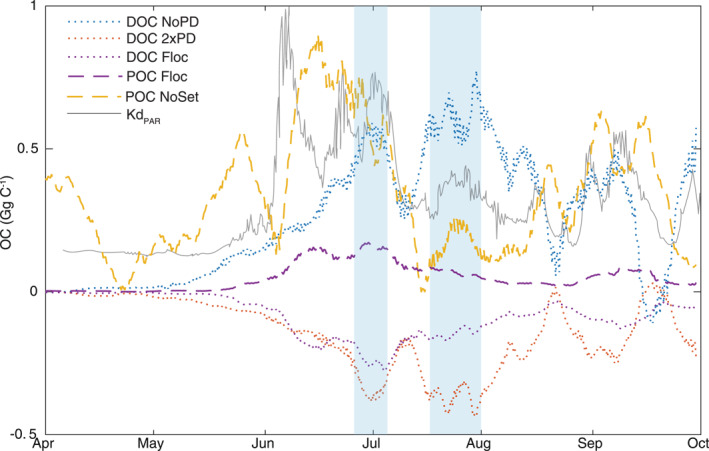
The difference from the baseline scenario of the fluxes of dissolved organic carbon (DOC) and particulate organic carbon (POC) across the plume transect for each of the scenarios, all smoothed with a 10‐day moving average. The gray line represents the vertically averaged and normalized (to a max of 4.4 m^−1^) diffuse attenuation coefficient (Kd_PAR_) for a plume location predicted by the radiative transfer module of YukonFVCOM‐ICM. The light‐blue shaded regions show two periods of highest difference between the NoPD and 2xPD scenarios.

The greatest difference in the NoPD and 2xPD DOC fluxes across the plume, from the baseline, occurred later in the summer peaking on 30 July (Figure 5). This is 1 month after the peak DOC flux of 30 June, indicating that residence time and recirculation within the plume enhances photodegradation. This is because as river flow decreases along with sediment supply, light penetration into the water column increases, which boosts photodegradation. This is supported by the time series of the vertically averaged diffuse attenuation coefficient of photosynthetically active radiation (PAR) (Kd_PAR_) calculated using the hyperspectral radiative transfer module within YukonFVCOM‐ICM (Figure 5). The maximum vertically averaged Kd_PAR_ occurred during the peak freshet when sediment and DOC export flux are greatest, and Kd_PAR_ decreased as discharge decreased and SPM settled out of the water column. Thus, there are two peaks when photodegradation impacted the plume DOC flux the greatest: the period of maximum DOC flux (30 June), when the rate of photodegradation is driven by the high concentration, which is then followed by the period in late July and August when CDOC concentrations remain relatively elevated within the plume waters but light penetration (the inverse of Kd_PAR_) was greatest allowing for more light‐activated photodegradation. Within the plume, the primary limiting factor of photodegradation is not incident irradiance (which occurs on 21 June with near 24‐hr sunlight, neglecting cloud and aerosol effects) but the light attenuation in the water column due to high levels of sediment during and after the spring freshet.

**Figure 6:**
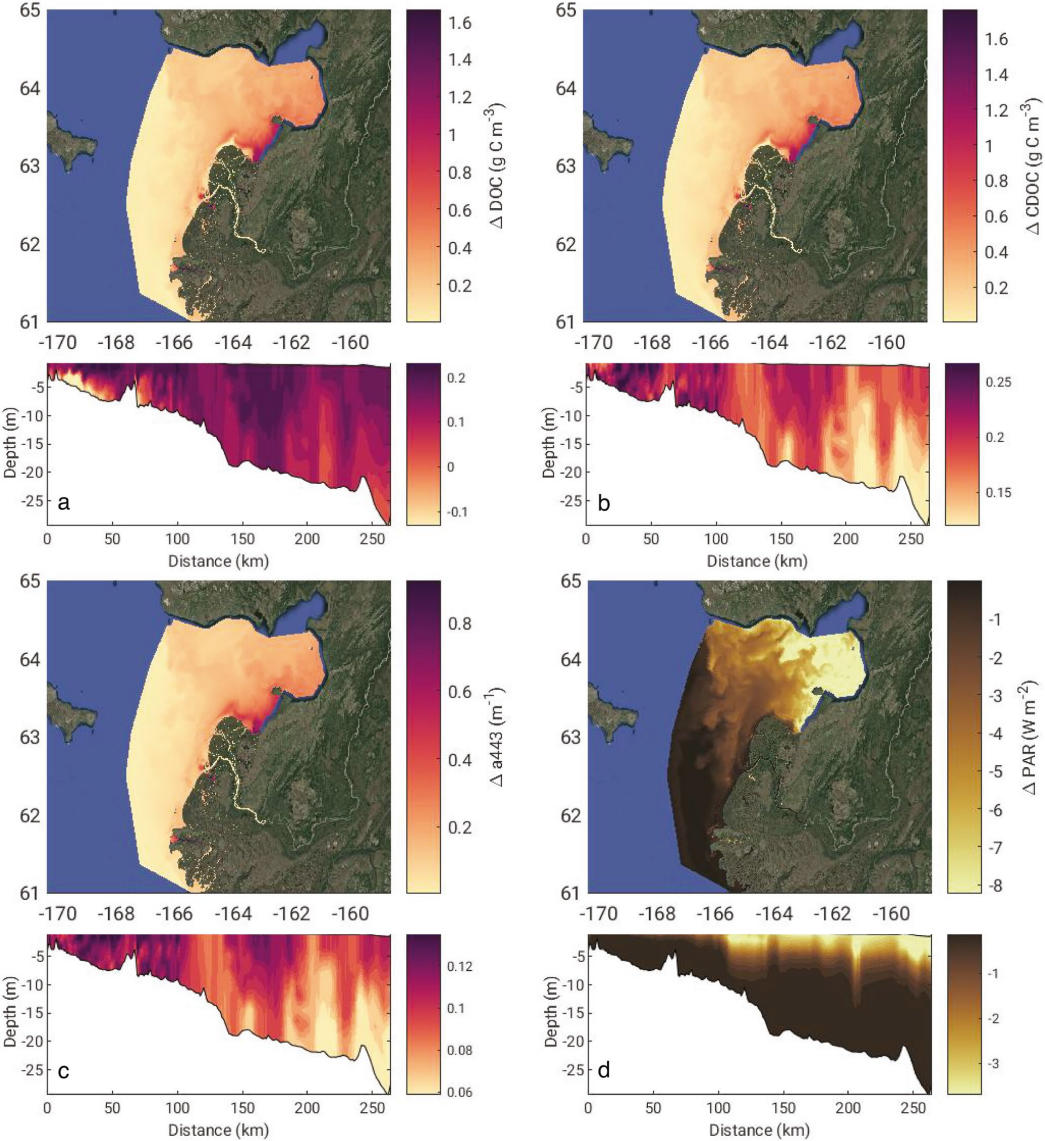
The difference between surface layer (a) dissolved organic carbon (DOC) concentration, (b) chromophoric DOC (CDOC) concentration, (c) chromophoric dissolved organic matter (CDOM) absorption at 443 nm, and (d) photosynthetically available radiation (PAR) between the no photodegradation and 2× photodegradation model scenarios (values = NoPD − 2xPD). The model fields were extracted on July 30 when the difference in the DOC flux across the plume between the two scenarios and the baseline was greatest. The surface plots are the surface layer of each model grid cell while the transect plots follow the western cruise track (Figure 1) from the river plume toward Nome, AK. The color shading was limited to the 95th percentile for each field.

Spatial plots and vertical transects extracted along the western cruise track from the Yukon River south mouth towards Nome, AK reveal the locations and depths of greatest difference in DOC, CDOC, and light field characteristics on 30 July (Figure 6). The greatest difference in all variables besides PAR occurred in the southeastern portion of Norton Sound just offshore of the North Mouth, likely a result of the predominant coastal currents pushing plume water from the south to the northeast where it circulates counter‐clockwise within Norton Sound (Clark & Mannino, 2022). Curiously, the NoPD versus 2xPD simulation generated less total DOC in the bottom waters at the beginning of the transect just outside of the south mouth (Figure 6a, lower panel). This phenomenon is likely due to elevated concentrations of DOC within the water column in the NoPD scenario depressing the sediment‐water column DOC flux that can make up a substantial contribution to estuarine and coastal organic carbon budgets (e.g., Burdige et al., 1992; Clark et al., 2017; Komada et al., 2013). The total difference in DOC mass within the model domain between the NoPD and 2xPD scenarios versus the Baseline on 30 July was 115 and −36.6 Gg C a potential difference in nearly 150 Gg C in the model domain that is attributed to photochemical reactions. Relative to the total average input from the Yukon River at Pilot Station of 1,300–1,500 Gg C DOC yr^−1^ (Holmes et al., 2012), photodegradation may play an important role in transforming terrestrial DOC in the ice‐free months in coastal waters, especially later in summer months.

The photodegradation scenarios led to the greatest differences in PAR occurring farther afield in Norton Sound than the other changes in the system (Figure 6d). Although Δ*a*_443_ is greatest at the north edge of the river delta (Figure 6c), the PAR differences are not realized until the impacts of suspended sediment on the underwater light field dissipate further offshore as sediment settled out of the water column. Once sediment has settled out of the water column 75–100 km down transect (Figure 6d), the impact of photodegradation on the PAR in the water column increased as CDOM remained elevated in the water column in the NoPD scenario. The photodegradation of CDOM impacts the underwater light availability further afield than riverine sediment inputs and can therefore exert some control on euphotic zone depth and phytoplankton growth in the coastal ocean. Accurate estimates of CDOM degradation rates and distribution in Arctic waters will lead to better predictions of future changes related to underwater light availability and phytoplankton production. Modeling analysis of Arctic phytoplankton production has shown that coastal inputs of nutrients and sediment can account for up to 1/3rd of phytoplankton production by the competing effects of light limitation and nutrient delivery (Terhaar et al., 2021). In addition, primary production has increased across the Arctic coinciding with the continuing loss of sea ice and increasing surface light availability (Lewis et al., 2020). If more CDOM is mobilized from land to ocean as runoff continues to increase and long stored carbon is mobilized into rivers, the underwater light availability for phytoplankton growth may be limited in nearshore regions. There are multiple possible feedbacks and having comprehensive cross‐season sampling across the plume‐ocean gradient of biogeochemical rates, light field characteristics, and primary production is key for an accurate estimate of coastal carbon cycling. This is because of the strong gradient in the dominant processes controlling underwater light distribution (and thus phytoplankton growth), photochemical processing of CDOM, and as the next section will show POC processes in the nearshore. The balance between increased light absorption by CDOM and decreased light absorption by sea ice may be a key feedback that could govern nearshore net carbon uptake and phytoplankton growth especially early in the ice‐free season.

#### 3.3.2 POC Settling

The scenario of decreased particle settling velocities to 0.0 m d^−1^ (NoSet) had very little change in the net flux of POC out of the river delta (Table 3). The impacts of the reduced settling are realized across the plume transect, where net total POC flux increased by 46.4% (61.3 Gg C), the majority of which was refractory RPOC (43.7 Gg C; 36.1%) and LPOC increased by 17.6 Gg C (161%). The NoSet flux was substantially greater than the Base scenario but 51% of riverine POC input was still lost within the plume. Between the hydrolysis of POC into DOC and the net settling of POC balanced by resuspension, it is difficult to ascertain what the predominant POC loss term is within the coastal waters. Further carbon accounting is necessary to better characterize the specific sources and sinks within the plume waters by integrating each POC biogeochemical process in time and space across the model domain. Phytoplankton predation and death likely contribute a substantial fraction to the net POC flux across the plume, in addition to sediment resuspension. These two sources can contribute very different POC reactivity classes and future biomarker analysis of collected suspended sediment and pigments will allow a more thorough characterization and model representation of POC sources and sinks within the plume. The hydrolysis rates of LPOC (0.03 d^−1^) and RPOC (0.006 d^1^) have an exponential decay half‐life of 23 and 116 days, respectively, suggesting significant hydrographic transport potential within the plume. A Lagrangian particle tracking exercise would be useful to characterize the average residence time of passive particles within the system. A large anticyclonic eddy from the south mouth that circulates plume waters back toward the coast and Norton sound is a dominant hydrographic feature (Clark & Mannino, 2022). This plume “bulge” increases retention in these nearshore waters allowing for hydrolytic breakdown of riverine derived POC to DOC, decreasing the net POC flux (greater breakdown) while increasing the net DOC flux out of the plume into Norton Sound.

**Figure 7:**
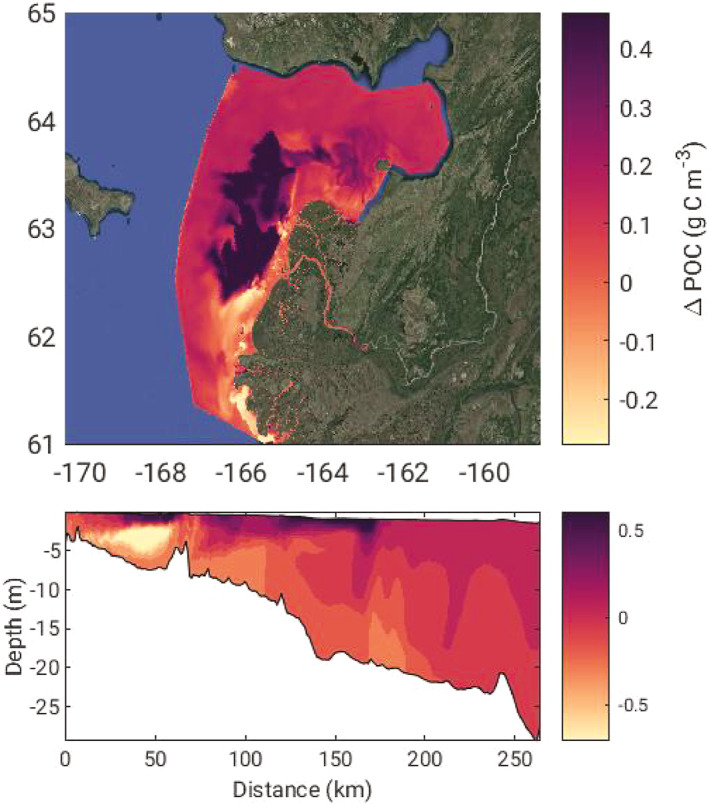
The difference in particulate organic carbon (POC) concentration from the no POC settling and the baseline scenario on June 16 when the difference between the POC flux of the two scenarios across the plume transect was greatest. The surface plots are the surface layer of each model grid cell while the transect plots follow the western cruise track (Figure 1) from the river plume toward Nome, AK. The color shading was limited to the 95th percentile for each field.

The greatest difference between POC flux (ΔPOC) in the NoSet and Base scenarios across the plume was 0.90 Gg C POC d^−1^ on 16 June (Figure 5), which was very close in time to the peak plume flux of POC (Figure 4b); the NoSet and Base difference closely tracked the river water flux patterns with primary and secondary peaks in POC flux difference and water discharge in June and late August‐September (Figure 5). The spatial patterns in the ΔPOC showed elevated POC concentration extending much further out into the surface waters away from the plume when settling velocity is 0.0 m d^−1^, with the area of greatest ΔPOC encompassing over 6,000 km^2^ (Figure 7). There was a decrease in POC in bottom waters nearshore within 50 km of the river mouth as POC remained suspended in the surface. These plots indicate the area of dominant settling is within 50 km offshore of the delta. Interestingly, there was greater POC in the surface waters in the baseline scenario south of the river, suggesting that some secondary interaction of POC sinking is supporting another source of POC such as phytoplankton growth and death in these waters. PON was treated similarly to POC, so the sinking of PON to the bottom with subsequent remineralization into NH_4_^+^ was also limited in the NoSet scenario. This may indicate that sedimentary PON hydrolysis and remineralization to NH_4_^+^ may be an important source of nitrogen for phytoplankton growth. There may also be a small effect on the underwater light availability, but the dominance of inorganic sediment (unchanged in the NoSet scenario) precludes POC impacts on light. At the peak ΔPOC flux difference, there was 38.8 Gg C POC stock (12.4%) more in the NoSet scenario which is 25.9% of the ΔDOC plume stock in the photodegradation scenarios.

#### 3.3.3 Flocculation

**Figure 8:**
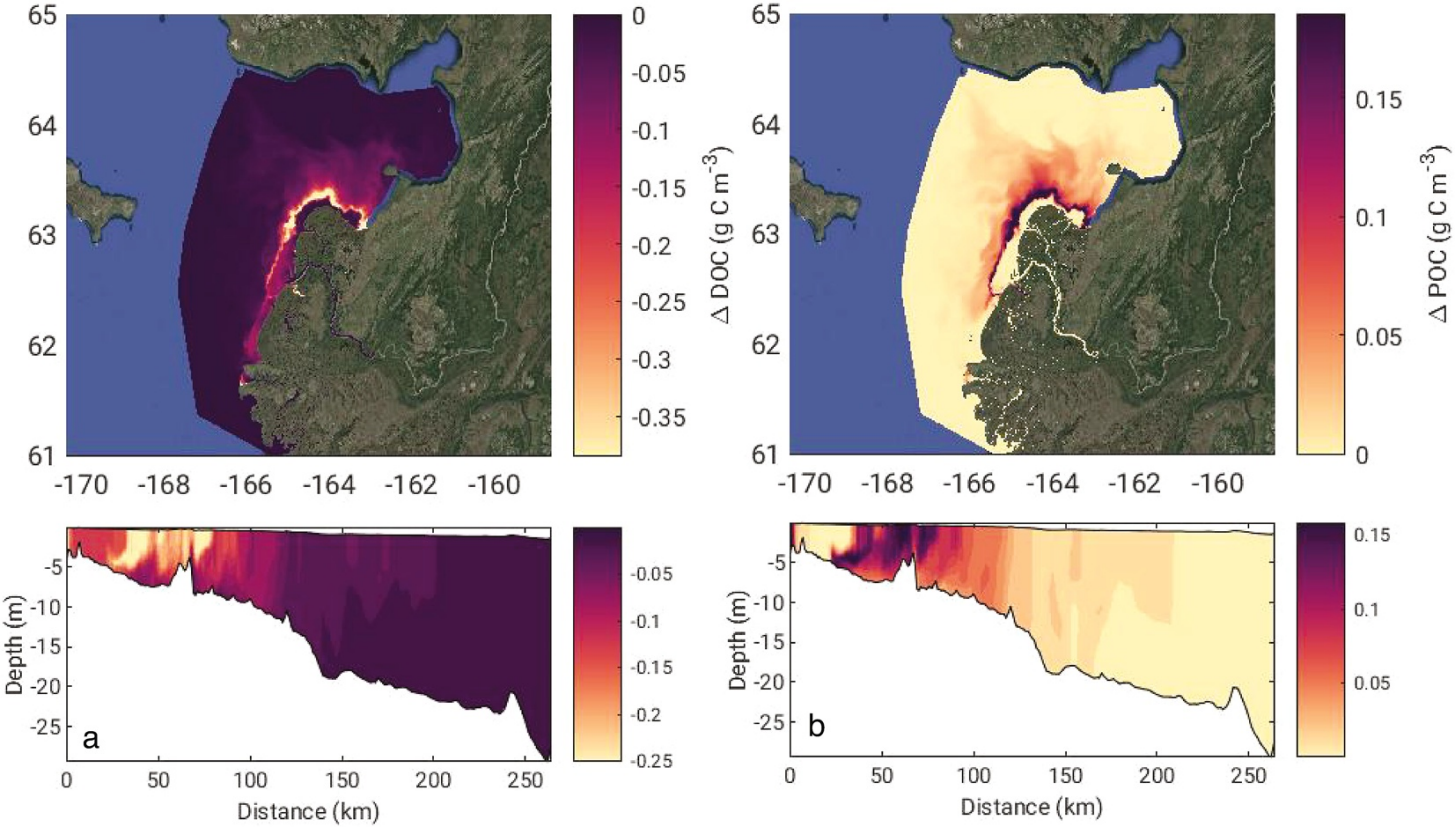
The difference between the Flocculation and Baseline scenarios for (a) dissolved organic carbon (DOC) and (b) particulate organic carbon (POC) on 4 July and 30 June when the difference in the flux across the plume transect was greatest for each variable, respectively. The surface plots are the surface layer of each model grid cell while the transect plots follow the western cruise track (Figure 1) from the river plume toward Nome, AK. The color shading was limited to the 95th percentile for each field.

Flocculation was modeled to represent the process of the binding of riverine‐derived DOC into POC upon freshwater mixing with saltier marine water. The impact of this initial first‐order flocculation process was assessed by including it as a final scenario where there were contrasting changes in the POC and DOC fluxes across the plume transect (Figure 5). There is a visible decrease in DOC and increase in POC in the nearshore region when the scenario is compared the baseline, with POC remaining elevated up to 100 km away from the delta (Figure 8). The total loss in organic carbon was only 6.3 Gg C (1.0%), with a 7% increase in POC and a 3% decrease in DOC, the majority of which was riverine DOC that was transformed into POC. While the 15.5 Gg C decrease in DOC flux and a 9.2 Gg C increase in POC flux out of the plume is relatively small, the *actual* kinetics related to POC‐DOC phase changes within these Arctic systems are still relatively unknown. Spencer et al. (2007) found that 0%–58% of DOC was removed during estuarine mixing in a temperate European river with a sharp drop relative to salinity, suggesting that the impact of flocculation can be substantially greater than what was estimated here. Understanding the specific type of DOC‐POC reactivity curves as they relate to each other in terms of concentration, salinity, and turbulence is key to having a better understanding of potential loss of DOC to POC and settling out of the water column into the sediment. While this initial scenario indicates that flocculation may be limited in importance in net organic carbon flux, a more detailed physico‐chemical experimental analysis is required to accurately represent flocculation kinetics in the YukonFVCOM‐ICM model.

### 3.4 Strengths and Areas to Improve the Modeling System

#### 3.4.1 Coastal Organic Carbon Process Modeling

The model was constructed and tuned to reproduce the spatial and temporal variability of DOC and POC, so it is not surprising that the observational comparison of the model output is quite strong. The unique ability to predict complex DOC processes such as hyperspectral light absorption and photochemical reactions is key for the transformation of DOC as it moves from terrestrial sources into the coastal ocean. Clearly, DOC and POC are complex mixtures, and therefore distilling them down into defined classes (six for DOC and two for POC) simplifies some of the inherent complexity in the environmental conditions. Particularly, phase change reactions (e.g., adsorption‐desorption and flocculation) and biological processes (e.g., uptake of POC and DOC into heterotrophic or mixotrophic plankton) are currently poorly constrained and represented in coastal models. In the future, we hope to utilize new experiments in the Yukon outflow and elsewhere in the Arctic to better constrain these processes so they may be included, if necessary, in the formulations for OC transformation. There is an inherent tradeoff between model complexity and tractability (Hood et al., 2006), but with careful observations we hope to further improve the model structure and parameterization. This will allow the explicit quantification of DOC‐POC interactions across the land‐ocean interface not only for the Yukon but other river‐influenced coastal systems in the Arctic. A nearby study from Kotzebue Sound found evidence for a large (40% ± 21%) terrestrial subsidy for benthic organisms that can play key roles in the coastal marine food web (McMahon et al., 2021). Therefore, having a realistic and useful modeling system is not only important for carbon budgeting but also for applications such as modeling higher trophic processes and culturally significant species dynamics that are already susceptible to the impacts of climate change (e.g., Grebmeier, 2012).

#### 3.4.2 Parameter Space Optimization and Model Temporal Expansion

We numerically optimized parameters related to the light field using a parameter space search algorithm within the ranges of in situ observational data (see Supporting Information S1). This allowed the best parameters related to the calculation of the underwater light field, a key environmental controlling factor for phytoplankton growth and photochemistry, to be implemented with confidence. Other parameters were extracted from the literature for Arctic systems (phytoplankton kinetics and limitation functions) and yet others were manually tuned to get the model to fit the data, especially for DOC, POC, and SPM concentrations across the model domain. Manual tuning of key parameters that requires expert model knowledge of controlling factors can potentially introduce bias and may limit the applicability of the model to other ecosystems, time periods, and user groups. With more observations across multiple years, we hope to undertake an un‐biased global parameter optimization for the key parameters identified here (Table 1), in addition to including results from experiments currently being conducted. This will allow for a more general optimal parameter set that can fit the model across years and environmental conditions, allowing extrapolation beyond the years where the bulk of the survey data was acquired.

#### 3.4.3 Phytoplankton Concentration and Growth From River to the Coast

While the model has little bias in predicting the mean phytoplankton chl *a* concentration, clearly there is difficulty in accurately predicting phytoplankton concentration across the delta‐plume‐ocean continuum (Figure S4 in Supporting Information S1). The current formulation only includes nitrogen limitation via a relatively complex NH_4_^+^ preference formulation (see Clark et al., 2020 for model equations). Other nutrient limitation formulations may be required, such as Si or Fe, to represent the phytoplankton growth functions more realistically within the overall model structure. A comprehensive model intercomparison exhibits the difficulty in predicting phytoplankton growth in the Arctic (Lee et al., 2015), and model complexity (i.e., by including more phytoplankton species) did not show a marked improvement over simpler formulations (Lee et al., 2015). Observations from the coast of the North American Arctic show that there is strong seasonal variability in both phytoplankton community composition and metabolism (Kellogg et al., 2019; Stoecker & Lavrentyev, 2018). The huge shift in underwater light availability from river to ocean also makes using a singular parameterization of phytoplankton light use efficiency and carbon to chl *a* ratios for the two phytoplankton groups too simple. Further observations of phytoplankton physiology in these polar coastal deltas and river plumes are necessary to constrain phytoplankton growth rates, community composition, and light and nutrient response to improve the model structure and parameterization. We are optimistic that with an enhanced phytoplankton growth representation, the modeling system can be used to understand linkages between riverine biogeochemistry and phytoplankton growth.

#### 3.4.4 Future Linkage to Satellite Remote Sensing

YukonFVCOM‐ICM contains a hyperspectral radiative transfer model that allows for the ability to predict hyperspectral remote sensing reflectance (Rrs). This is currently done offline using the regional model parameterization of IOPs (Supporting Information Methods and Figure S2 in Supporting Information S1) and the Ocean‐Atmosphere Spectral Irradiance Model (OASIM; Gregg & Casey, 2009). Preliminary results show good matchups with in situ measured hyperspectral remote sensing reflectance (Rrs), and future work will provide a direct linkage with satellite‐observed ocean color properties. This allows for another method of model validation and the potential to link model processes such as DOC degradation and phytoplankton production to *observed* ocean color. This also provides a unique test bed for predicting hyperspectral Rrs in a complex coastal environment in preparation for future satellites such as the Plankton, Aerosol, Cloud, ocean Ecosystem (PACE) that will carry the hyperspectral Ocean Color Instrument (OCI). Combining processed based modeling capability with multi‐ and hyperspectral Rrs has potential to better quantify carbon flow in coastal systems through model‐satellite data combination and assimilation (IOCCG, 2020).

## 4 Conclusions

Overall, YukonFVCOM‐ICM accurately represented the spatial and temporal variability of surface DOC and POC in the spring freshet of 2019. Most of the river organic carbon flux exited the river delta through the southern mouth with minimal loss before the delta and less than half of the organic carbon was transported away from the coast and out of the plume toward the Bering Strait via coastal currents. Microbial degradation of DOC was the largest loss term within the plume, and photodegradation decreased the flux of DOC out of the plume by 9.6% indicating a potential important role even in relatively turbid coastal waters. The DOC flux decreased mainly because of the photodegradation of biologically recalcitrant DOC into the more reactive DOC classes, not direct photomineralization. The interplay between photodegradation, microbial DOC remineralization, and primary production governs the net DOC flux into the ocean, and presumably the exchange of CO_2_ in surface waters. The interaction of phytoplankton production and microbial degradation has recently been shown to be the dominant process of CO_2_ evasion in two Alaskan streams (Rocher‐Ros et al., 2021), and YukonFVCOM‐ICM indicates the same is likely in the Yukon River plume.

The impact of photodegradation on the DOC pool and the underwater light field was clearly observed throughout the model domain in the NoPD versus 2xPD scenario ΔDOC plots (Figure 6), and photodegradation decreased the total DOC stock in the model domain by 115 Gg C at peak ΔDOC on 30 July. Photodegradation also substantially decreased the offshore transport of CDOM which allowed for an increase in underwater light availability and primary production away from the plume. POC made up a smaller portion of the OC flux and stock in the coastal ocean and settling accounted for 46% of the loss of POC from the river out of the plume transect. While POC sinking and resuspension dynamics are extremely complex, the simple formulations employed here allowed for a relatively accurate prediction of the POC and SPM in time and space. Further experimental and observational research into POC and DOC chemical properties and interactions across seasons will allow for a more refined model parameterization and the potential for predictions of the state of the Yukon River and coastal ocean into the future. Finally, future inclusion of the inorganic carbon cycle and carbonate chemistry (Bianucci et al., 2018) into YukonFVCOM‐ICM will give us the ability to compose a near‐complete carbon budget across the land‐ocean continuum for this complex coastal environment. Realistic representation of the carbon cycle in the Arctic and coupling this coastal modeling system with other models of ice, erosion, and terrestrial runoff and permafrost thaw can give us the ability to represent the climate‐carbon feedbacks in the coastal Arctic accurately now and into the future.

## Conflict of Interest

The authors declare no conflicts of interest relevant to this study.

## Supporting information

Supporting Information S1

## Data Availability

The model inputs and outputs for this research are available on the NASA NCCS public server at https://portal.nccs.nasa.gov/datashare/yukonriver/Carbon, the model validation data used is available at the citations listed in the NASA SeaBASS archives under project ARCTIC_RSWQ. The source code for the ICM‐DOM‐PD model is available at https://github.com/bclark805 and with https://doi.org/10.5281/zenodo.7293688 (bclark805, 2022).
