# Supplementary material for: The Transformation and Export of Organic Carbon Across an Arctic River‐Delta‐Ocean Continuum
Source: J Geophys Res Biogeosci. 2022 Dec 12;127(12):e2022JG007139. doi: 10.1029/2022JG007139 (PMC10078588; doi:10.1029/2022JG007139)
Supplement: Supplementary file 1 — Supporting Information S1 [file JGRG-127-0-s001.pdf]

AGU PUBLICATIONS

Journal of Geophysical Research: Biogeosciences

Supporting Information for

The Transformation and Export of Organic Carbon Across an Arctic River-Delta-Ocean Continuum

J. Blake Clark[1,2], Antonio Mannino[1], Maria Tzortziou[3], Robert G.M. Spencer[4], and Peter Hernes[5]

1. Ocean Ecology Laboratory, Code 616.1, NASA Goddard Space Flight Center, Greenbelt, MD, USA

2. Goddard Earth Sciences Technology and Research II, University of Maryland, Baltimore County, Baltimore, MD, USA

3. Department of Earth and Atmospheric Sciences, The City College of New York, The City University of New York, New York, NY, USA

4. Department of Earth, Ocean and Atmospheric Science, Florida State University, Tallahassee, FL, USA

5. Department of Land, Air and Water Resources, University of California, Davis, CA USA.

Contents of this file

Text S1 to S2.3
Figures S1 to S7

Introduction

Below is supporting information for the above manuscript. There is text that describes in detail the methodology used to derive the inherent optical property spectra used in the bio-optical model with the YukonFVCOM-ICM system. There are also figures related to the model validation for secondary variables not directly related to the manuscript outcomes or conclusions.

S1. Numerical Optimization of Hyperspectral Inherent Optical Properties

To derive estimates and uncertainty of IOPs in the river plume, an iterative model optimization scheme was constructed in MATLAB using the Ocean Atmosphere Spectral Irradiance Model (OASIM; Gregg and Casey 2009) to predict Rrs. The model was forced with estimated daily time series of optically active constituents that were constructed from comprehensive observations by the Arctic Great Rivers Observatory (Holmes et al. 2020). The model was iterated 1 million times for each 183 day "model year" with varying magnitude and shape of IOP mass specific absorption and scattering spectra that were bounded by observations collected in the river and river plume in 2019. The model was optimized to recreate hyperspectral observed Rrs (sr^{-1}) collected in the river plume in June of 2019 (Mannino et al. 2021). The optimal spectra were found by minimizing the sum of the square residuals (SSR) across the predicted (183 million in total) vs. observed Rrs Spectra and the coinciding IOP absorption spectra and constituent concentration were identified. Finally, a first cut at algorithm development to estimate model predicted CDOM absorption at 355 nm, a_{355} (m^{-1}) in these highly turbid waters were assessed to get a better understanding of the possibility of estimating CDOM and DOC from hyperspectral Rrs observations on the water and from space.

S1.1 Radiative Transfer Modeling of Remote Sensing Reflectance

To calculate the light propagation, attenuation, and reflectance in the water column, the optical constituent absorption and scattering properties are utilized in a set of numerical equations in the Ocean Atmosphere Spectral Irradiance Model (OASIM; Gregg and Casey 2009) framework. First, the surface downwelling irradiance ($I_0(\lambda)$; W m^{-2}), is calculated from the UV-Visible portion of the NARR downwelling short-wave irradiance by taking 43% of the total NARR irradiance and multiplying by the spectral distribution of light at Earth's surface. OASIM is a well characterized spectral radiative transfer model designed for simulating downwelling irradiance and upwelling radiance in the ocean and atmosphere (Gregg and Casey 2009). OASIM calculates the time-variant three-way radiative transfer of downwelling irradiance ($E_d(\lambda)$; W m^{-2}), downwelling diffuse irradiance ($E_s(\lambda)$ W m^{-2}) and upwelling radiance ($E_u(\lambda)$; W m^{-2}) (Gregg and Casey 2009; Gregg and Rousseaux

2017). By factoring the mean cosines for each direction, and various shape factors depending on the direction of backscatter (Ackleson et al. 1994), the normalized water leaving irradiance, $L_wN(\lambda)$, is calculated (Aas 1987). Finally, to predict hyperspectral remote sensing reflectance (Rrs(λ); sr^{-1}), $L_wN(\lambda)$ is divided by $I_0(\lambda)$. A thorough description of OASIM can be found in Gregg and Casey (2009) and Gregg and Rousseaux (2017).

S2.1 Initial Estimates of Inherent Optical Properties

In OASIM, the absorption and scattering of the individual optically active constituents are needed to calculate the total absorption and scattering, which is then input into the three-stream radiative transfer model (Gregg and Casey, 2009). Total absorption, $a_t(\lambda)$ (m^{-1}), is calculated as the sum of the absorption due to water, $a_w(\lambda)$, absorption due to suspended, depigmented particles (detritus), $a_{SPM}(\lambda)$, absorption due to CDOM, $a_{CDOM}(\lambda)$, and the absorption due to phytoplankton, $a\varphi(\lambda)$, all having units of m^{-1} (Eq. S1). The absorption due to pure water, $a_w(\lambda)$, was specified using the most recent estimates from the IOCCG Protocol Series (2018) and was interpolated to the model wavelengths of 285:700 at 5 nm intervals using a piecewise cubic Hermite interpolation (PCHIP). The spectral distribution of light was estimated from the downwelling irradiance sensor from the Hyperpro-SBA that was deployed in the Yukon plume to measure hyperspectral surface optical properties (Mannino et al. 2021). The mean spectral irradiance was calculated and interpolated to the model wavelength interval (5 nm) and range (285-700 nm) and the irradiance at each wavelength was then divided by the integrated irradiance to yield the fractional irradiance for each wavelength.

$$a_t(\lambda) = a_w(\lambda) + a_{SPM}(\lambda) + a_{cdom}(\lambda) + a_\varphi(\lambda) \qquad \text{(S1)}$$

$$a_C(\lambda) = a^*C(\lambda)[C] \qquad \text{(S2)}$$

$$bb_t(\lambda) = bb_w(\lambda) + bb_p(\lambda) \qquad \text{(S3)}$$

$$bb_p(\lambda) = bb^*p(\lambda)[SPM] \qquad \text{(S4)}$$

S2.2 Calculating Individual Absorption and Scattering Spectra

Each optically active constituent requires a mass-specific absorption spectra, a*C(λ), that yields the spectral absorption by that constituent, $a_C(\lambda)$, when multiplied by the constituent concentration, [C] (CDOC, SPM, chl *a*) (Eq. S2). Particle specific absorption, $a^*_{SPM}(\lambda)$, was calculated by dividing coincident measurements of particle absorption, $a_{SPM}(\lambda)$, by the mass of SPM (Fig. S2a) and the range was calculated as the mean±σ (Fig. S2a). The range of a*CDOC specific absorption was calculated from the Arctic-GRO measurements at Pilot Station, AK, as described below and the mean and standard deviation (σ) of each set of $a^*_{CDOC}(\lambda)$ were calculated and the mean±σ was set as the range of which the $a^*_{CDOC}(\lambda)$ varied for each model iteration (Fig. S2a).

The treatment for the absorption of phytoplankton chl *a* was slightly different than CDOC and SPM because of the difficulty in accurately measuring *in-situ* pigment absorption because of the high sediment concentrations in the river and plume stations. Chl *a*nd other pigments concentration were measured via high performance liquid chromatography, and pigments were used qualitatively to assess what phytoplankton likely dominate in the sampled areas (Dr. Aimee Neeley, pers. comm.). The dominant phytoplankton absorption spectra were then assigned using the measured absorption and chl *a* concentration from axenic cultures (Lomas et al. *in prep*) and averaged to yield one a*φ(λ) to be used in Eq. S2 (Fig. S2a). Chl *a* concentration was not measured at Pilot Station, AK as the other optically active constituents, so at each day in each model iteration chl *a* was randomly specified between 2.97±1.95 μg L^{-1} (mean±σ) of observations from the river plume stations collected in 2019 (Mannino and Novak, 2021). For each model iteration, the values of $a^*_{CDOCW}(\lambda)$, $a^*_{CDOCSS}\lambda)$, and $a^*_{SPM}(\lambda)$ were randomly varied within their range to yield a single value which when multiplied by the forcing concentration and combined with the daily randomized chl *a* concentration $a_w(\lambda)$ yielded the $a_t(\lambda)$.

The total backscatter coefficient, $bb_t(\lambda)$ (m^{-1}) (Eq. S3), is the other IOP that is needed in OASIM to calculate radiative transfer. While many types of particles can contribute $bb_t(\lambda)$, in this study, particles were not divided into individual components. This was done because of how SPM is measured (gravimetrically) and because $bb_t(\lambda)$ is measured *in-situ* and not on individual scattering components, although the *in-situ* backscatter due to water, $bb_w(\lambda)$, is removed from the signal. $bb_w(\lambda)$ is calculated at each

time step in the model from the specified river temperature and salinity=3 using the Zhang et al. (2009) formulations. To calculate the mass specific particle scattering spectra, $bb^*_p(\lambda)$, observations of $bb_p(\lambda)$ from the river plume and coastal ocean were divided by coincident measurements of SPM to yield the mean±σ $bb^*_p(\lambda)$ (Fig. S2b).

Each mass specific spectra needed a value at 5 nm intervals between 285-700 nm for every model run, and for the absorption spectra the measurements cover the entire range necessary for the model. $bb^*_t(\lambda)$ was necessarily treated differently because it was only measured at 9 wavelengths from 409-715 nm; for each model iteration, the value at the 9 measurement wavelengths was randomly specified within the mean±σ to yield 9 independently varying spectral values. This treatment allows for numerous potential spectral shapes which coincides with the measurements that lacked a defined spectral shape and didn't follow the classical power law typically specific for particle backscattering spectra. Between measurement wavelengths and beyond 409 nm into the UV values were interpolated and extrapolated using the PCHIP methodology, and below 409 nm the extrapolated spectra were bounded by the minimum and maximum of the mean±σ for all measured wavelengths.

S2.3 Model Optimization Scheme

An untargeted stochastic optimization scheme was employed to find the best mass specific absorption and backscatter coefficient spectra that matched the OASIM predicted Rrs(λ) with the observed Rrs(λ) from the Yukon River plume collected in 2019. First, the mean and standard deviation for each $a^*(\lambda)$ and $bb^*_p(\lambda)$ were calculated to yield a range of potential values. Initially, the mean for each was used, and the 183-day simulation was run to obtain Rrs(λ) for each day. This was considered the baseline run and served as a reference point that the model would be improved upon. Next, the model was set to iteratively run to minimize a model-skill cost function, the sum of the square of residuals (SSR) (Eq. S5) (Stow et al. 2009). The model was set up so each randomized (within range) spectra were specified at the start of each iteration, then the 183-day simulation was conducted using the estimated river input time-series described above. After the time-series simulation, Rrs(λ) for each day was compared to the mean SBA Rrs(λ) and the best (least) SSR from the simulation was found. Model time points where SSR was less than 9×10^{-5} sr^{-1} were identified, and Rrs and associated concentrations and IOPs were extracted. In a test

model run of 100k simulations 9×10^{-5} was the threshold for the top 5% of simulations. For the final optimization, 1 million simulations were conducted which took ~6 hours on a 32 GB 2017 MacBook Pro.

$$SSR = \sum [Pred - Obs]^2 \quad \text{(S5)}$$

Figures

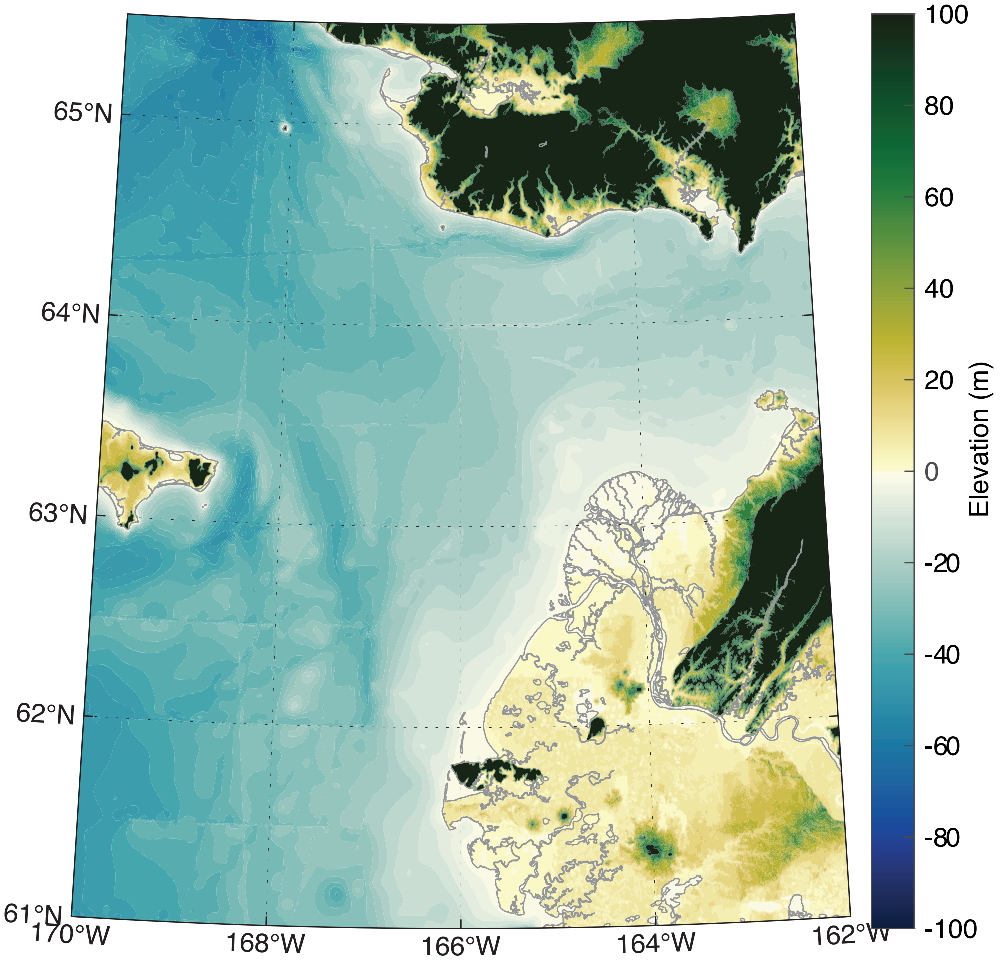

Figure S1 The northern Bering Sea and lower Yukon River and delta elevation above sea level and bathymetry.

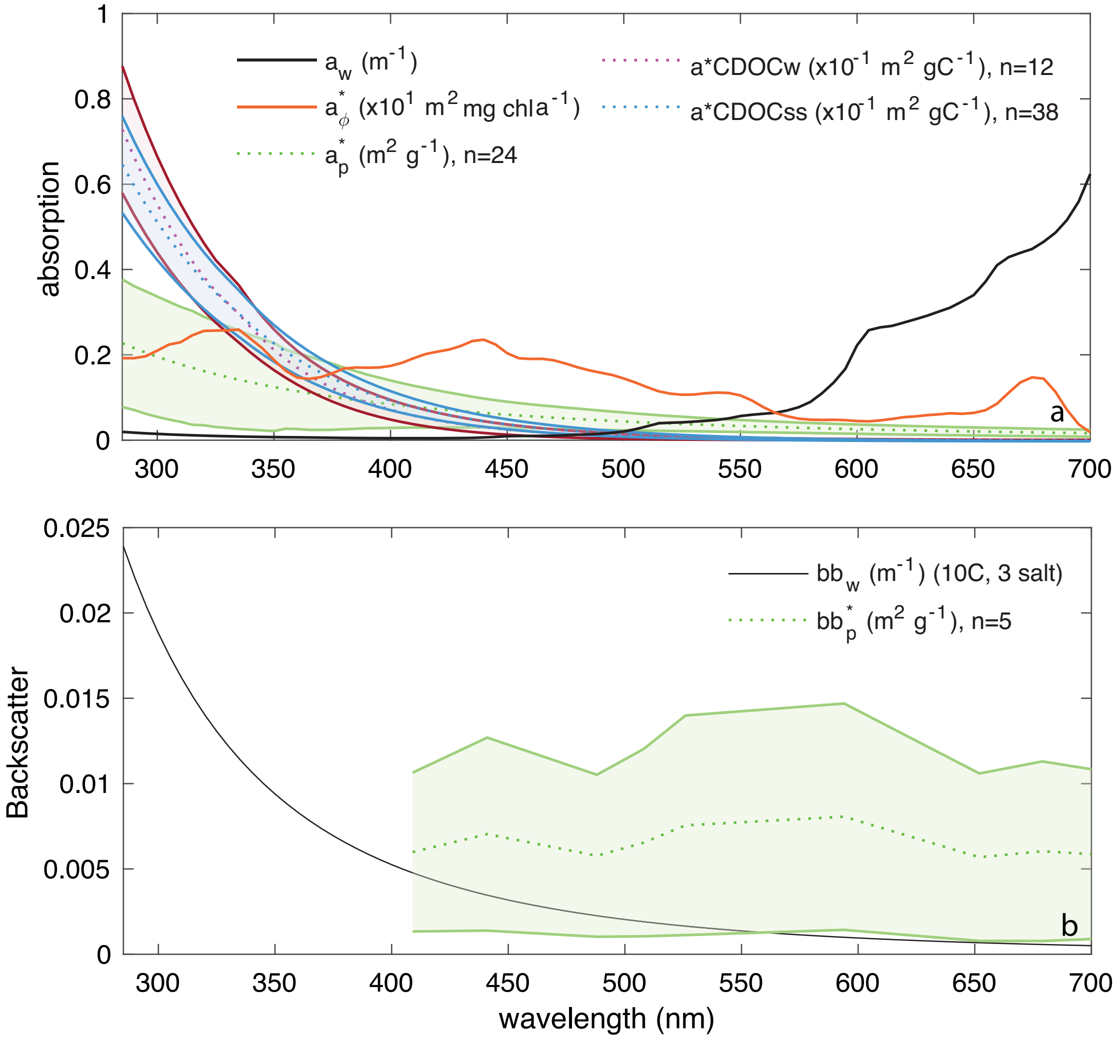

Figure S2 (a) The absorption due to water, a_w, chl *a* mass- specific absorption, a^*_φ, non-algal particle absorption, a^*_{SPM}, and the winter and spring/summer colored dissolved organic carbon mass specific absorption spectra, a^*CDOC_W & a^*CDOC_{SS} and (b) backscatter coefficient due to water, bb_w, and particle mass specific backscatter coefficient, bb^*_p, (upper panel) and Solid lines around bb^*_p a^*CDOC_W, a^*CDOC_{SS}, and a^*_{SPM} are the range of the spectra used in the optimization scheme.

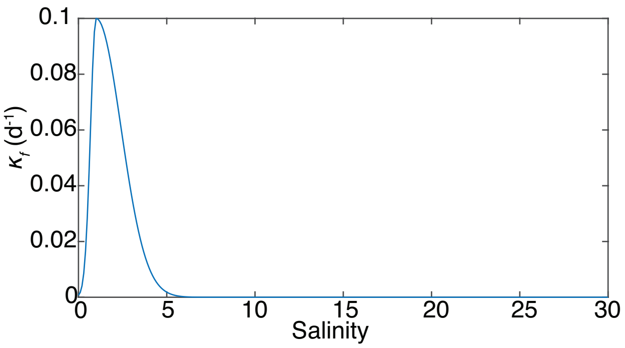

Figure S3 The rate of flocculation κ_f as a function of salinity utilized in the Flocculation scenario.

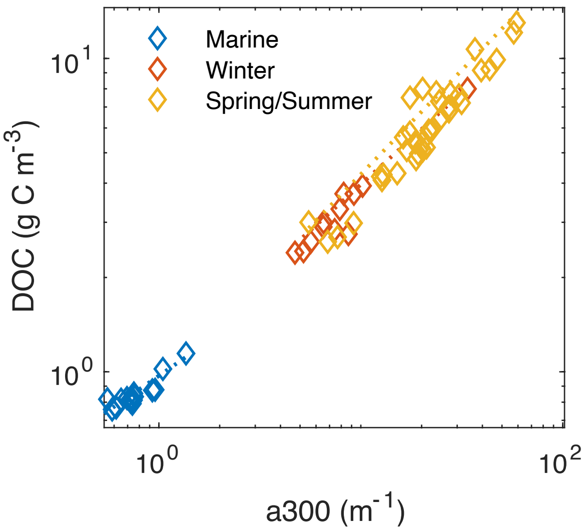

Figure S4 Dissolved organic carbon (DOC) as a function of CDOM absorption at 300 nm (a300) used to define the marine, winter riverine and spring/summer riverine colored DOC pools. The linear model fits were all significant with R^2 of 0.9, 0.96, and 0.91 for marine, winter, and spring/summer respectively. Winter was delineated as the months before May and after October following Holmes et al. (2012).

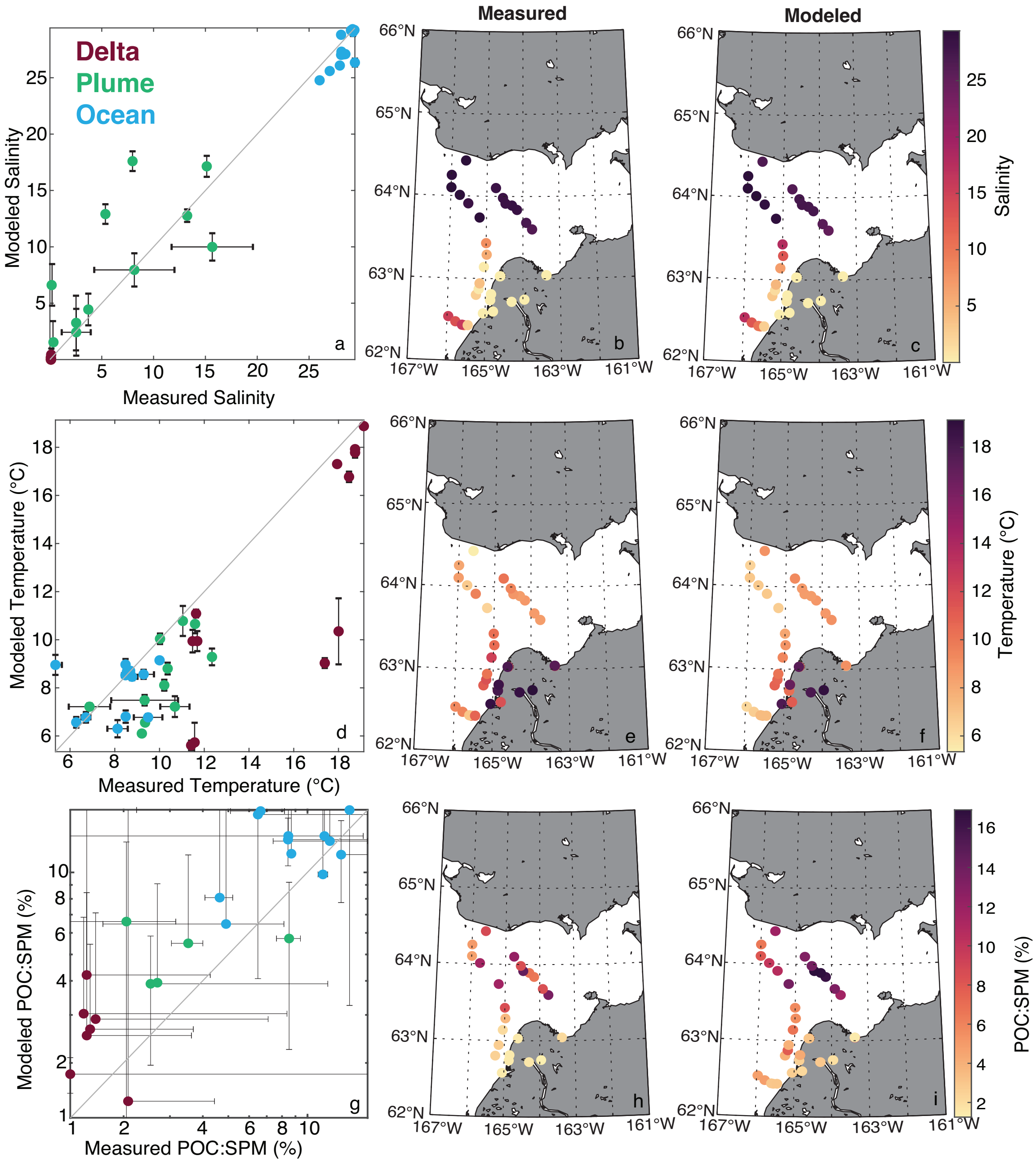

Figure S5 Model data comparisons for surface salinity (upper), temperature (middle) and the particulate organic carbon (POC) percentage of suspended particulate matter (SPM; lower). The error bars represent the ± 1 standard deviation of the measurements of the model output over a 36 hour period bracketing the best matchup in time and space.

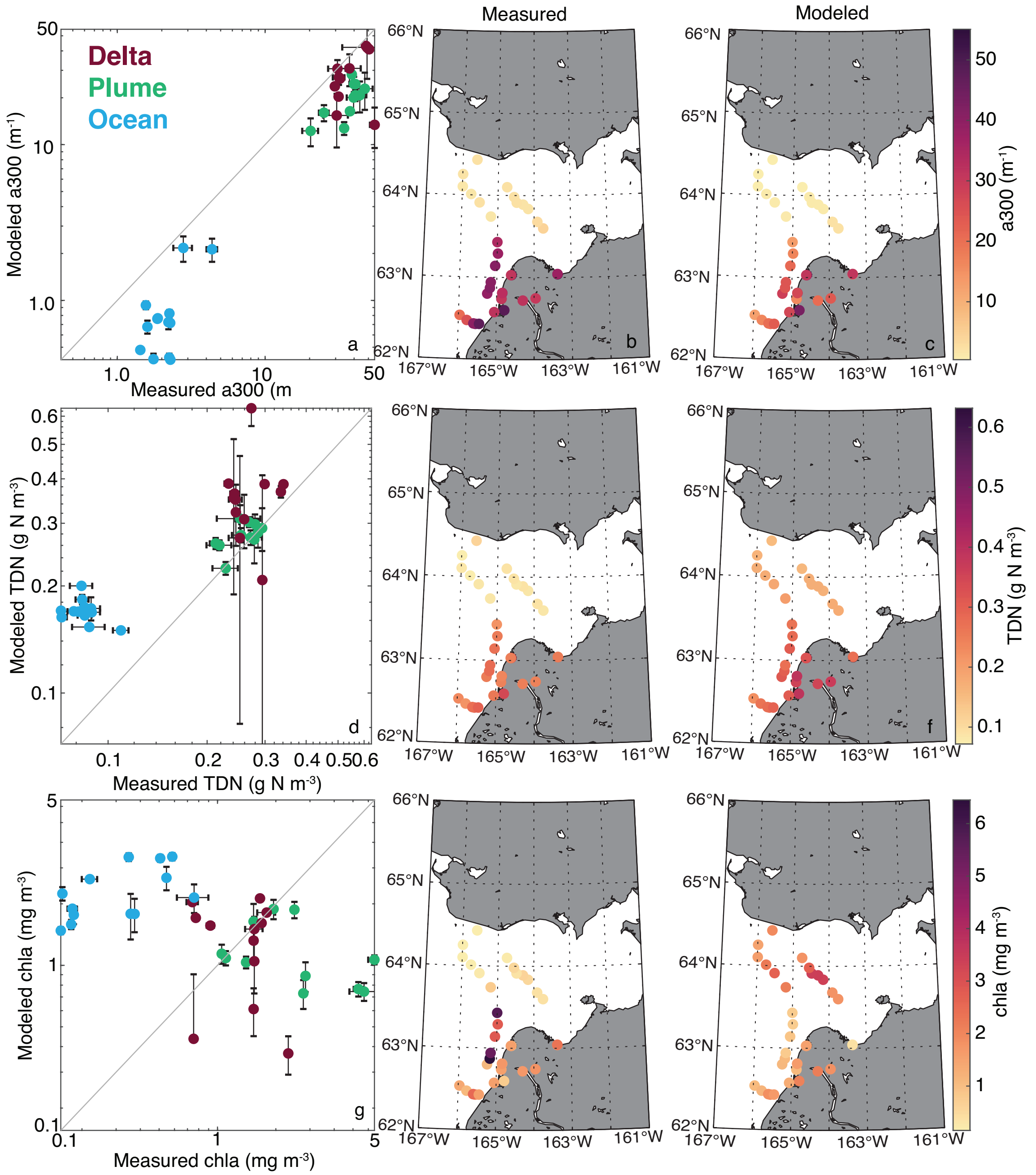

Figure S6 Model data comparisons for surface colored dissolved organic matter (CDOM) absorption at 300 nm (a300; upper), total dissolved nitrogen (TDN; middle) and chl *a* concentration (chl *a*; lower). The error bars represent the ± 1 standard deviation of the measurements of the model output over a 36 hour period bracketing the best matchup in time and space

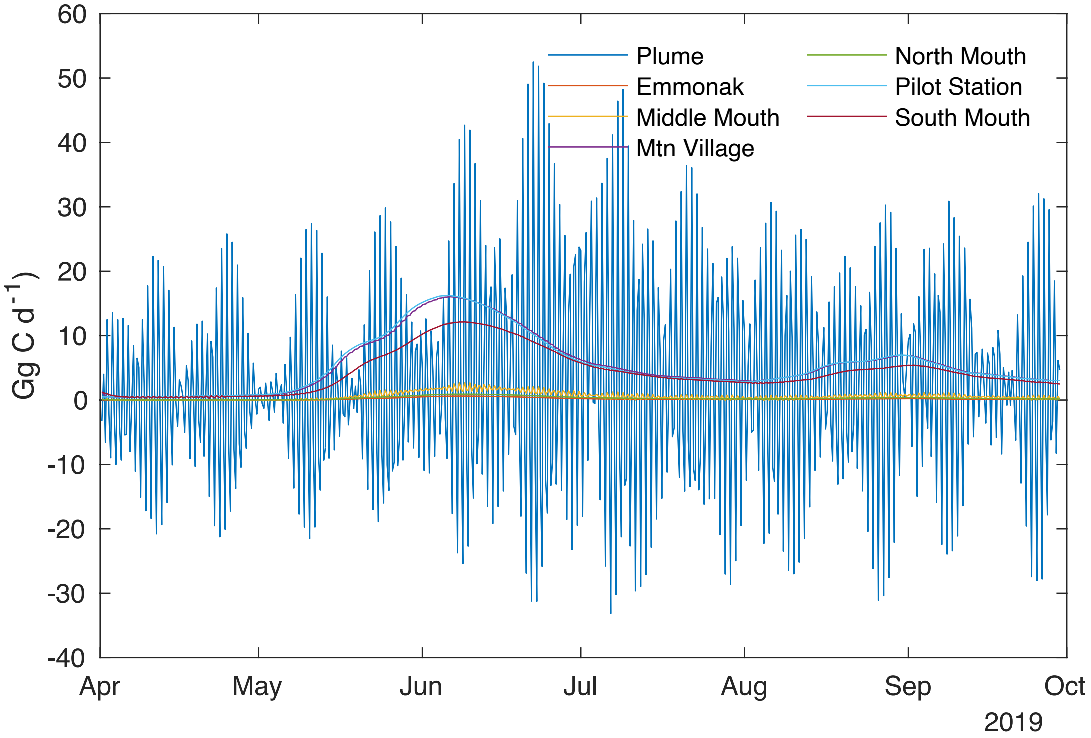

Figure S7 The unfiltered dissolved organic carbon (DOC) flux across each of the transects shown in Fig. 1 in the main text showing the tidal variability across the plume.
